# Transcriptome-wide identification and characterization of miRNAs from *Pinus densata*

**DOI:** 10.1186/1471-2164-13-132

**Published:** 2012-04-06

**Authors:** Li-Chuan Wan, Haiyan Zhang, Shanfa Lu, Liang Zhang, Zongbo Qiu, Yuanyuan Zhao, Qing-Yin Zeng, Jinxing Lin

**Affiliations:** 1 Key Laboratory of Plant Molecular Physiology, Institute of Botany, Chinese Academy of Sciences, Beijing 100093, China; 2 Medicinal Plant Cultivation Research Center, Institute of Medicinal Plant Development, Chinese Academy of Medical Sciences & Peking Union Medical College, Beijing, Haidian District 100193, China; 3 Graduate School of the Chinese Academy of Sciences, Beijing 100049, China; 4 State Key Laboratory of Systematic and Evolutionary Botany, Institute of Botany, Chinese Academy of Sciences, Beijing 100093, China

**Keywords:** *Pinus densata*, miRNA, Transcriptome

## Abstract

**Background:**

MicroRNAs (miRNAs) play key roles in diverse developmental processes, nutrient homeostasis and responses to biotic and abiotic stresses. The biogenesis and regulatory functions of miRNAs have been intensively studied in model angiosperms, such as *Arabidopsis thaliana*, *Oryza sativa *and *Populus trichocarpa*. However, global identification of *Pinus densata *miRNAs has not been reported in previous research.

**Results:**

Here, we report the identification of 34 conserved miRNAs belonging to 25 miRNA families from a *P. densata *mRNA transcriptome database using local BLAST and MIREAP programs. The primary and/or precursor sequences of 29 miRNAs were further confirmed by RT-PCR amplification and subsequent sequencing. The average value of the minimal folding free energy indexes of the 34 miRNA precursors was 0.92. Nineteen (58%) mature miRNAs began with a 5' terminal uridine residue. Analysis of miRNA precursors showed that 19 mature miRNAs were novel members of 14 conserved miRNA families, of which 17 miRNAs were further validated by subcloning and sequencing. Using real-time quantitative RT-PCR, we found that the expression levels of 7 miRNAs were more than 2-fold higher in needles than in stems. In addition, 72 *P. densata *mRNAs were predicted to be targets of 25 miRNA families. Four target genes, including a nodal modulator 1-like protein gene, two GRAS family transcription factor protein genes and one histone deacetylase gene, were experimentally verified to be the targets of 3 *P. densata *miRNAs, pde-miR162a, pde-miR171a and pde-miR482a, respectively.

**Conclusions:**

This study led to the discovery of 34 conserved miRNAs comprising 25 miRNA families from *Pinus densata*. These results lay a solid foundation for further studying the regulative roles of miRNAs in the development, growth and responses to environmental stresses in *P. densata*.

## Background

MicroRNAs (miRNAs), generally 21-24 nt in length, spatiotemporally regulate gene expression at transcriptional and/or posttranscriptional level in most eukaryotes [1]. They play important roles in plant development, nutrient homeostasis, responses to biotic and abiotic stresses and antibacterial reactions [2-5]. Most miRNAs are transcribed by RNA polymerase II (Pol II) from intergenic regions [6]. Like messenger RNAs (mRNAs), primary miRNA (pri-miRNA) transcripts possess 5' caps and 3' poly(A) tails [7]. Pri-miRNAs are processed into imperfect hairpin precursor miRNAs (pre-miRNAs) and then double-stranded miRNA:miRNA* duplexes by Dicer-like 1 (DCL1) protein [8,9]. The duplexes are exported into the cytoplasm by HASTY. One selected strand of the duplexes (mature miRNA) is recruited by the ARGONAUTE1 (AGO1) protein to form the so-called RNA-induced silencing complex (RISC) [10]. Mature miRNAs guide the complexes to target mRNAs by base complementarity for direct cleavage or translational repression [11].

Identification of conserved and novel miRNAs usually relies on two approaches: bioinformatic prediction and experimental sequencing. *In silico *prediction of miRNAs includes searching genomic or EST databases for orthologous sequences of known miRNAs and analyzing their pre-miRNA hairpin structures [12]. The limitation of this approach is that only highly conserved miRNAs can be identified. The advent of high-throughput sequencing technologies, such as massively parallel signature sequencing (MPSS), 454 and sequencing-by-synthesis (SBS), has greatly accelerated the discovery of medium-to-low abundant and species-specific miRNAs from diverse plants, e.g., *Triticum aestivum*, tomato and *Oryza sativa *[13-25]. For plant species with complete genome information, conserved and novel miRNAs can be conveniently identified based on their alignments to the genome and known miRNAs in the miRBase and analysis of their pre-miRNA stem-loop structures.

Hundreds of miRNAs have been identified and characterized in model angiosperms, such as *Arabidopsis*, rice and poplar [26-29]. However, few reports involving miRNAs in conifers and other gymnosperms exist. A total of 37 miRNAs from the stem xylem of *Pinus taeda *have been identified and the expression of 10 miRNA families was significantly repressed in the galled stem [30]. Stage-specific modulation of specific miRNAs and miRNA biogenesis components in zygotic embryos and female gametophytes of *P. taeda *were demonstrated to play vital roles during embryogenesis and seed development [31]. Morin et al. reported 53 candidate novel miRNA families from *Pinus contorta *[32]. By sequencing of small RNA libraries constructed from a *Taxus chinensis *cell line, Qiu et al. found that the expression levels of 14 miRNAs were down-regulated whereas that of 3 miRNAs were up-regulated after treatment with methyl jasmonate [33]. In *Pinus abies*, 24 novel and 4 conserved miRNAs were identified, and 7 conserved and 9 novel miRNAs were found participating in epigenetic regulation [34].

*Pinus densata *is an ecologically important conifer. It represents a highly successful case of homoploid hybrid speciation with far-reaching evolutionary consequences [35]. But to date, little is known about its transcriptional sequence information. Global identification of *Pinus densata *miRNAs has not been reported in previous research. To gain the knowledge of its mRNA transcriptome, we recently performed a high-throughput sequencing of mRNAs isolated from *P. densata *needles. The present study was designed to identify *P. densata *miRNAs from the mRNA transcriptome database. Subcloning and sequencing were conducted to further confirm the pri- and/or pre-miRNA sequences. Meanwhile, using real-time RT-PCR, the expression profiles of 10 miRNAs in *P. densata *seedling tissues were examined. We have also predicted miRNA targets and 4 target mRNAs were experimentally validated by 5' RACE. Our study is the first comprehensive investigation of miRNAs in *P. densata*, which not only forms a solid base for further study of regulative functions of miRNAs in the development and growth, but also expands our knowledge of conifer miRNAs.

## Results

### Transcriptome-wide survey of miRNAs in *P. densata*

**Figure 1:**
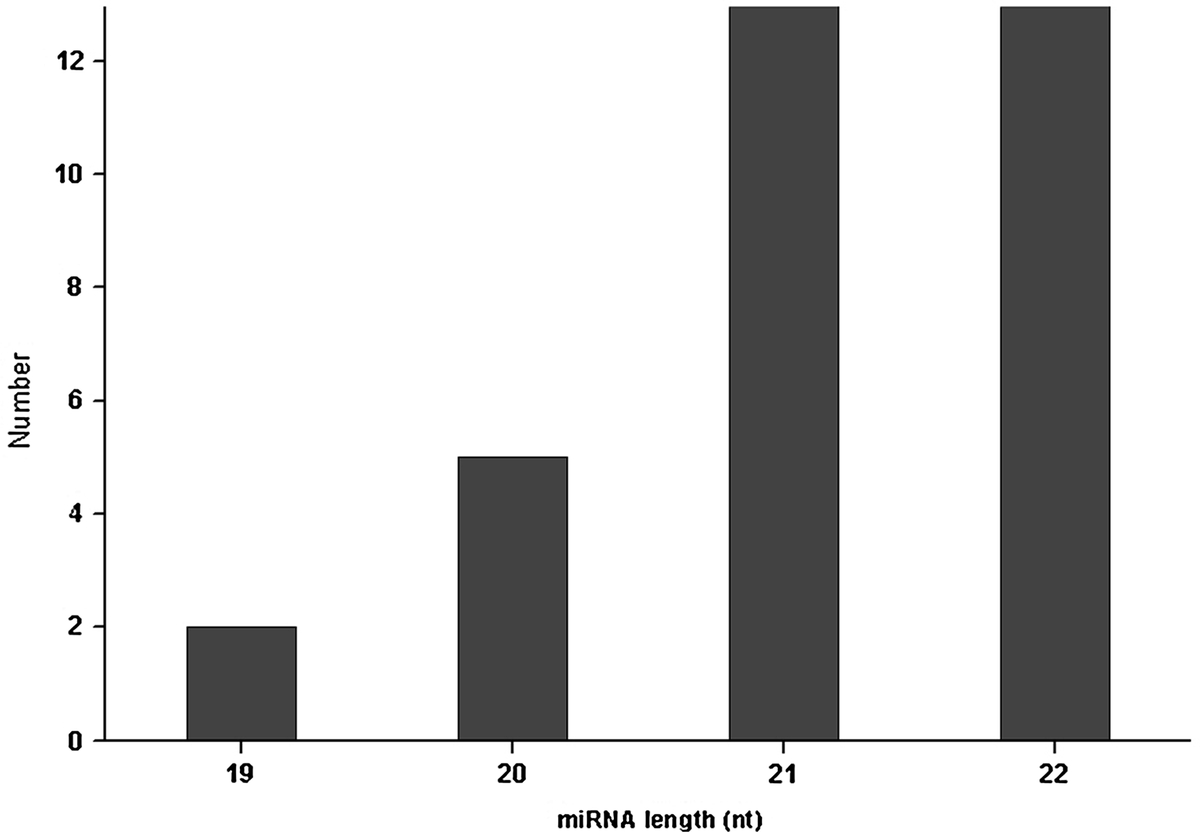
**MiRNA length distribution**.

Using Illumina sequencing technology, we obtained 3,968,794 raw sequences and 84,950 consensus-genes, including 287 clusters and 84,663 singletons, from a *P. densata *mRNA library. To identify conserved miRNAs, we used 3,968,794 sequences as query against mature and precursor sequences in the public microRNA database (miRBase version 16), which contains 2952 miRNAs across 43 plant species [36]. A total of 34 conserved miRNAs were identified comprising 25 miRNA families. The sequences of mature, pre- and pri-miRNAs are shown in Table 1 Additional file 1 and Additional file 2 respectively. The length of *P. densata *miRNA precursors ranged from 76 to 526 nt, with a majority of them (80%) ranging from 67 to 150 nt. It is consistent with that observed in *Arabidopsis *and rice [37]. The minimal folding free energy indices (MFEIs) of *P. densata *miRNA precursors varied from 0.54 to 1.28, with an average value of 0.92. It is similar to that of other plant miRNAs, such as *Arabidopsis*, rice, *Glycine max*, *Medicago truncatula*, *Saccharum officinarum*, *Sorghum bicolor *and *Zea mays *[38]. The hairpin structures of *P. densata *miRNA precursors predicted by MFOLD are shown in Additional file 3 and Additional file 4. The length of *P. densata *mature miRNAs varied from 19 to 22 nt, with 21 (38%) and 22 (41%) nt ones as the two major size classes (Figure 1). Notably, 19 (58%) miRNAs start with a 5' terminal uridine residue, a characteristic feature of miRNAs recognized by the AGO1 protein. These results imply that the identified *P. densata *miRNAs may be canonical.

**Table 1:** Conserved miRNAs identified in *P.densata*

miRNA gene	miRNA sequence (5'-3')	Arm	Length (nt)	A + U (%)	Folding energy	MFEI	RT-PCR	qPCR	*Conserved in other plants					
									
									ath	osa	ptc	vvi	pta	pab
pde-miR159a	UUUGGUUUGAAGGGAGCUCUA^a^	3'	21	53.5	-94.74	0.89	+	+	+	+	+	+	+	
pde-miR162a	UCGAUAAACCUCUGCAUCCAG	3'	21	45.0	-49.10	0.80			++	+	++	++		
pde-miR166a	UCGGACCAGGCUUCAUUCC	3'	19	45.8	-49.10	0.96	+	+	+	+	+	++	+	+
pde-miR166b	UCGGACCAGGCUUCAUUCC	3'	19	48.8	-43.40	1.01	+	+	+	+	+	++	+	+
pde-miR169a	CAGCCAAGGAUGACUUGCCUA^a^	5'	21	58.3	-48.80	1.14	+		+	+	+	+		
pde-miR171a	UGAUUGAGCCGUGCCAAUAUC	3'	21	55.2	-55.20	1.28	+	+	+	++	++	++	+	
pde-miR390a	AAGCCCAGGAUGGAUAGCGCC	5'	21	53.7	-40.50	0.92		+	+	+	+	+	++	
pde-miR396a	UCCCACGGCUUUCUUGAACUU^a^	5'	21	55.1	-43.28	0.90			+	+	+	+	+	+
pde-miR482a	UCUUUCCUACUCCUCCCAUUCC	3'	22	52.3	-60.90	0.98	+	+	+		+	++	+	++
pde-miR482b	UCUUCCCUAUUCCUCCCAUUCC	3'	22	52.1	-60.30	1.04	+		+		+	+	+	++
pde-miR482c	GGCUUGCGAGGGUAGGAAAAG^a^	5'	21	48.9	-45.20	0.90			+		+	+	+	+
pde-miR482d	CCUUUCCAACGCCUCCCAUGCC^a^	3'	22	54.8	-46.50	0.76	+		+		+	+	+	+
pde-miR783	AUUCUUUGCUGGUUCAUUUUC	3'	21	57.0	-26.80	0.67	+						+	
pde-miR946a	CAGCCCUUCUCCUAUCCACAA	3'	21	59.3	-71.50	1.02	+	+					++	
pde-miR947	CAUCGGAAUCUGUUACUGUUUC	3'	22	48.7	-70.70	0.94	+						++	+
pde-miR949a	UCUCUAGGAAUCAAAUGUGUC^a^	5'	21	47.7	-41.80	0.91	+						+	
pde-miR949b	UCUCCGGGAAUCCAAUGCGCC	5'	21	46.3	-66.30	1.12	+						++	
pde-miR950a	UCUGGUCCACGGUGGUUUAU^a^	5'	20	57.2	-40.90	1.05	+	+					+	+
pde-miR951	UGUUCUUGACGUCUGGACCAC	5'	21	54.8	-43.00	0.83	+						++	+
pde-miR952a	AACAGAGCAUGCCAUUGGUG^a^	5'	20	54.0	-232.79	0.96	+						++	
pde-miR952b	AACAGAGCAUGCCAUUGGUG^a^	5'	20	53.9	-214.40	0.99	+						++	
pde-miR952c	AACAGAACAUGCCAUUGGUG^a^	5'	20	54.2	-192.12	0.90	+						++	
pde-miR1310	GGCAUCGGGGGCGUAACGCCCU	5'	22	47.0	-35.00	0.80	+						++	
pde-miR1311	UCAGAGUUUUGCCAGUUCCGCC	3'	22	48.8	-43.40	0.99	+	+					++	++
pde-miR1312a	UUUGGAGAGAAAAUGGCGACAU	3'	22	62.8	-41.50	0.81	+						++	
pde-miR1313	UACCACUGAAAUUGUUGUUCG^a^	5'	21	58.6	-66.72	0.71	+	+					+	
pde-miR1314a	CCGGCCUCGAAUGUUAGGAGAA^a^	3'	22	56.2	-42.30	0.92	+	+					+	
pde-miR1448	CUUUCCAACGCCUCCCAUGC^a^	3'	20	54.8	-46.50	0.76	+				+			
pde-miR2118a	UUUCCAACGCCUCCCAUGCCUA^a^	3'	22	54.8	-46.50	0.76	+			+				
pde-miR2118b	UUCCCUAUUCCUCCCAUUCCUA^a^	3'	22	49.4	-42.00	0.98	+			+				
pde-miR3701	UGAACAAUGCCCACCCUUCAUC^a^	3'	22	59.3	-84.10	1.07	+							+
pde-miR3704a	GGUCUCGGUGGAGUUGGGAAGA^a^	5'	22	53.8	-49.00	0.98	+							+
pde-miR3704b	GGUCUCGAUGGAGUUGGGAAGA^a^	5'	22	54.7	-46.40	0.95	+							+
pde-miR3712	UGUGAUCAAGAUCAGACUCCCA^a^	5'	22	59.4	-15.00	0.54	+							+
						0.92 ± 0.15^b^								

*Ath, osa, ptc, vvi, pta and pab are the abbreviations for *A. thaliana*, *O. sativa*, *P. trichocarpa*, *Vitis vinifera*, *P. taeda *and *P. abies*, respectively. The plus symbols indicate: ++, miRNAs identical to known miRNAs in other plant species; +, miRNAs homologous to known miRNAs in other plant species. ^a ^is a novel member of corresponding miRNA family. ^b ^0.92 ± 0.15 is the average and standard deviation of MFEI values of pre-miRNAs.

**Figure 2:**
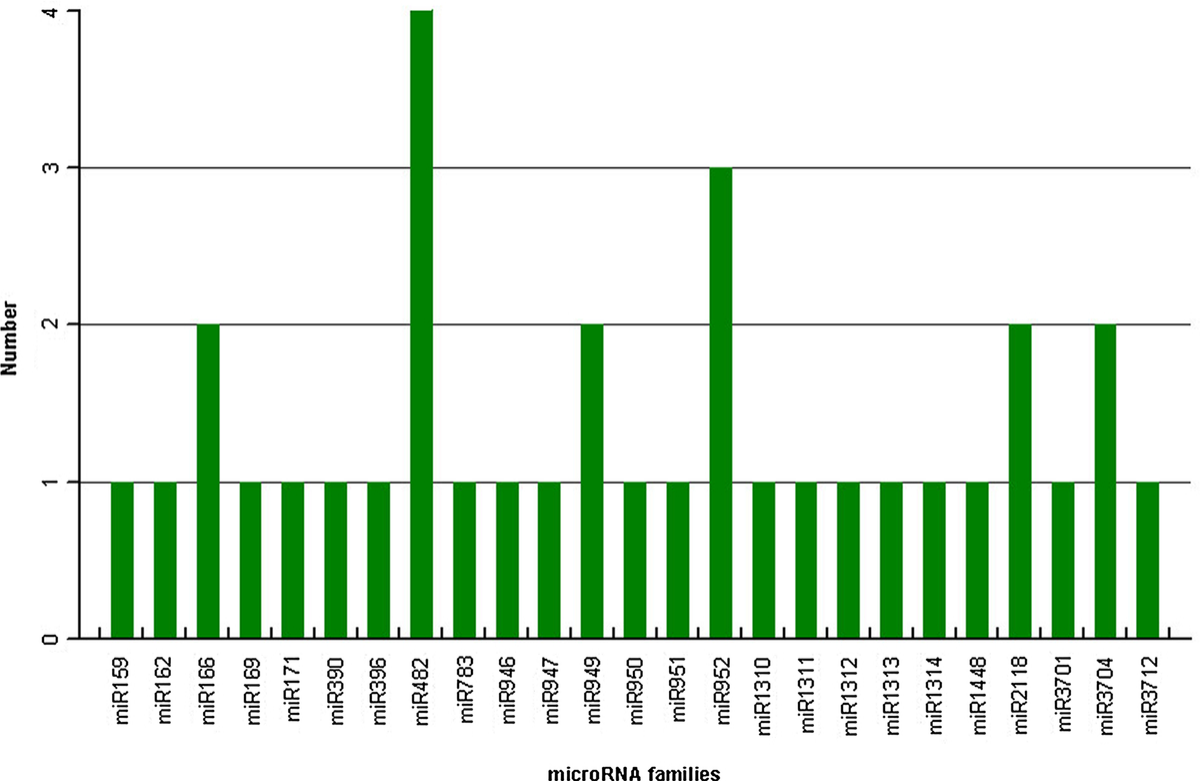
**Numbers of miRNA family members in *Pinus densata***.

Sequences of miRNAs within a family are identical or nearly identical and mismatched nucleotides between two miRNA family members are up to 4 [39]. Through the analysis of the 34 *P. densata *miRNA precursors, we identified 19 novel mature miRNAs belonging to 14 conserved miRNA families. It includes pde-miR159a, pde-miR169a, pde-miR396a, pde-miR482c, pde-miR482d, pde-miR949a, pde-miR950a, pde-miR952a, pde-miR952b, pde-miR952c, pde-miR1313, pde-miR1314a, pde-miR1448, pde-miR2118a, pde-miR2118b, pde-miR3701, pde-miR3704a, pde-miR3704b and pde-miR3712 (Table 1), of which 17 miRNAs were further validated by subcloning and sequencing except pde-miR396a and pde-miR482c. Interestingly, each miRNA family contains diverse members in *P. densata *(Figure 2). For example, the pde-MIR482 family has 4 members, whereas only one exists in 19 miRNA families (pde-MIR159, pde-MIR162, pde-MIR169, pde-MIR171, pde-MIR390, pde-MIR396, pde-MIR783, pde-MIR946, pde-MIR947, pde-MIR950, pde-MIR951, pde-MIR1310, pde-MIR1311, pde-MIR1312, pde-MIR1313, pde-MIR1314, pde-MIR1448, pde-MIR3701 and pde-MIR3712).

We identified several singleton sequences from the *P. densata *mRNA transcriptome database highly homologous with the precursors of *P. taeda *or *P. abies *miR948, miR1309, miR1315, miR1316 and miR3700. In addition, seven contig sequences identical to or highly homologous to osa-miR156k, osa-miR399a, osa-miR414, pta-miR948, cre-miR1171, pta-miR1309 and pta-miR1316 were also found in the database (Additional file 5). However, they were not able to form canonical secondary hairpin structures. Therefore, we excluded them from the miRNA candidate list.

In this study, we have tried to identify novel miRNAs in *P. densata*. Because of the lack of sRNA database of *P. densata*, 166 and 191 small RNAs from *Pinus taeda *and *Picea abies*, two evolutionally related species from Pinaceae, were downloaded and used in the analysis as described above [30,34]. No novel miRNA was found, which could be attributed to insufficient small RNAs and the limited number of mRNAs in the *P. densata *transcriptome database.

### Validation of pri- and pre-miRNA sequences

We carried out subcloning experiments to validate the pri- and pre-miRNA sequences. The pri-miRNA sequences of 25 miRNAs, including pde-miR159a, pde-miR166a, pde-miR166b, pde-miR169a, pde-miR171a, pde-miR482a, pde-miR482b, pde-miR482d, pde-miR783, pde-miR946a, pde-miR947, pde-miR949a, pde-miR949b, pde-miR951, pde-miR952a, pde-miR1310, pde-miR1311, pde-miR1312a, pde-miR1313, pde-miR1448, pde-miR2118a, pde-miR2118b, pde-miR3701, pde-miR3704a and pde-mi3712, and the pre-miRNA sequence of pde-miR950 were experimentally confirmed. Three novel pri-miRNA sequences, pde-miR952b, pde-miR952c and pde-miR3704b were identified when sequencing the clones for pde-miR952a and pde-miR3704a. Seven of the 29 validated sequences were identical to the sequences obtained from Illumina sequencing, whereas 18 of which had less than 10 mismatched nucleotides and 4 of which had more than 10 mismatched nucleotides, which might be partially attributed to sequence assembly mistakes during the Illumina sequencing. The pre-miRNA sequences of 5 miRNAs, pde-miR162a, pde-miR390a, pde-miR396a, pde-miR482c and pde-miR1314a could not be amplified from total RNAs of two-month-old seedling stems, although we repeated our experiments. It could be due to their tissue-specific expressions or limited information for primer design.

**Figure 3:**
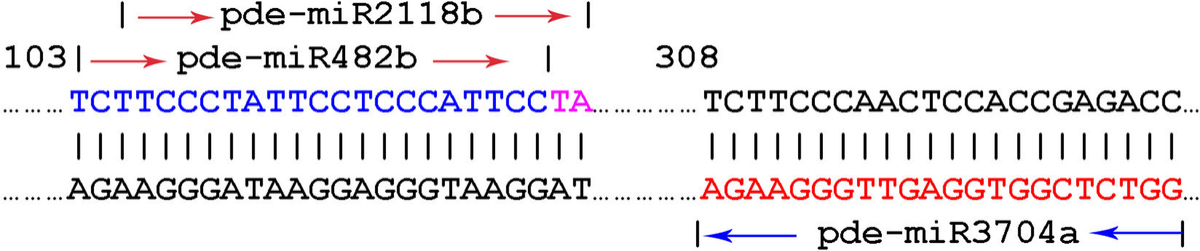
**Pde-miR482b, pde-miR2118b and pde-miR3704a clustered in a contig**. The sense and antisense strand of singletons868998 harbor mature sequences of pde-miR482b, pde-miR2118b and pde-miR3704a.

Intriguingly, the precursor sequences of pde-miR482b and pde-miR2118b were found clustered in a single contig (singletons868998), while the antisense strand of which harbored the precursor sequence of pde-miR3704a (Figure 3). Since the primary sequences of pde-miR482b and pde-miR3704a were confirmed, it might be reasonable to deduce that the three miRNA precursors were genuine precursors. Singletons3959581 was also predicted containing precursor sequences of 3 miRNAs, pde-miR482d, pde-miR1448 and pde-miR2118a. Its sequence was validated by both Illumina sequencing and subcloning. Similar phenomenon was also reported in *P. taeda *[30].

### Expression patterns of miRNAs

**Figure 4:**
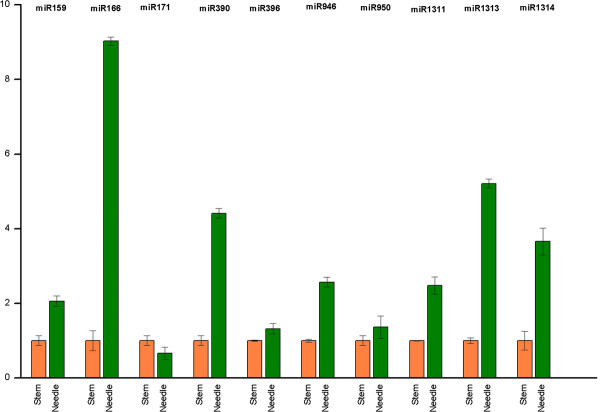
**Transcript profiles of *P. densata *conserved miRNAs**. Samples are needles and stems of 2-month-old seedlings. The expression levels of all miRNAs by real-time PCR were relative to that of 5 S rRNA and normalized. Error bars represent the standard deviations of three PCR replicates of a single reverse transcription reaction. The normalized miRNA levels in stems were arbitrarily set to 1.

In order to obtain solid evidence to support the existence and expression of conserved miRNAs in *P. densata*, we examined the expression profiles of 10 mature miRNAs (pde-miR159a, pde-miR166a, pde-miR171a, pde-miR390a, pde-miR396a, pde-miR946, pde-miR950, pde-miR1311, pde-miR1313 and pde-miR1314b) in needles and stems of two-month-old seedlings, using real-time RT-PCR (Figure 4). Our results demonstrated that 9 of them had higher expression levels in needles than in stems except pde-miR171. The expression levels of 7 miRNAs, including pde-miR159a, pde-miR166a, pde-miR390a, pde-miR946, pde-miR1311, pde-miR1313 and pde-miR1314b, were more than 2-fold higher in needles than in stems, Intriguingly, miR166a, an important miRNA known for the functions in establishment of adaxial/abaxial (dorsoventral) leaf polarity, was expressed more than 9 times higher in needles than in stems [40]. The result suggests that pde-miR166 may play key roles in a variety of physiological processes in *P. densata *needles.

### Prediction of miRNA targets

To better understand the functions of *P. densata *miRNAs, we predicted their targets using the Web-based program psRNATarget http://bioinfo3.noble.org/psRNATarget/index.php?function=function3[41]. A total of 3,968,794 sequences from the *P. densata *mRNA transcriptome database and 38 mature miRNAs were used as a custom target database and a custom miRNA database respectively. Seventy-two mRNAs were predicted to be targets of 25 miRNA families (Table 2 and Additional file 6), of which 10 (14%) targets were homologous to previously validated or predicted miRNA targets in *A. thaliana*, *O. sativa*, *P. trichocarpa*, *P. taeda*, *P. abies *and/or *T. chinensis *(Table 2). It includes DCL1 targeted by pde-miR162, GRAS family transcription factor cleaved by pde-miR171, Class III HD-Zip protein HDZ33 regulated by pde-miR166 and CC-NBS-LRR resistance-like protein sliced by pde-miR2118. Searching target enrichment in the gene ontology (GO) http://www.geneontology.org/ showed that these conserved targets were involved in a variety of physiological processes in plants. The number of conserved targets of each miRNA family varied from 1 to 3. Pde-MIR2118 family had three conserved targets. Pde-MIR171 family had two conserved targets, while pde-MIR162 and pde-MIR166 families each had only one conserved target.

**Table 2:** Conserved miRNA targets and their putative functions

miRNA	Target function	Target^a^	*Conserved with						GO annotation
				
			ath	osa	ptc	pta	tch	pab	
pde-miR162	DCL1	Singletons83286 (2)	+	+	-	-	-	-	RNA processing
	Nodal modulator 1-like	Singletons11093 (1) ^b^	+	+	+	-	-	-	Carboxypeptidase activity
pde-miR166	Class III HD-Zip protein HDZ33	Singletons59617 (2)	+	+	-	-	-	+	DNA binding
pde-miR171	GRAS family transcription factor	Singletons10015 (0.5)^b^ Singletons84522 (0.5)	+	+	-	-	-	-	DNA binding
	Actin binding protein	Singletons83401 (3) ^b^	-	-	-	-	-	-	Actin binding
pde-miR482	Histone deacetylase	Singletons7264 (3) ^b^	+	+	+	-	-	+	Histone deacetylation
pde-miR2118	CC-NBS-LRR resistance-like protein	Singletons50083 (3) Singletons65538 (3) Singletons72472 (3)	-	+	-	-	-	+	Defense response

^a ^All predicted miRNA targets with penalty scores (shown in parentheses) of three or less are listed.

^b ^validated by RLM-5' RACE.

*Ath, osa, ptc, pta, tch and pab are the abbreviations for *A. thaliana*, *O. sativa*, *P. trichocarpa*, *P. taeda*, *T. chinensis *and *P. abies*, respectively.

The other 62 mRNAs targeted by 22 *P. densata *miRNA families showed no similarity to other plant miRNA targets. These targets were predicted to play essential roles in multiple physiological processes. The CC-NBS-LRR resistance-like protein and disease resistance protein targeted by pde-MIR2118 family miRNAs appeared to be involved in defense response. Other putative targets include anion exchanger family protein, ATP binding protein and chaperone ClpB. Forty-two predicted target mRNAs encode hypothetical or unknown function proteins. No targets were found for 5 miRNA families, including pde-MIR390, pde-MIR1310, pde-MIR1311, pde-MIR3701 and pde-MIR3704. It could be due to insufficient mRNAs in the mRNA transcriptome database.

### Validation of miRNA-guided cleavage of mRNA

Mature miRNAs can direct RISC complexes to slice target mRNAs or inhibit their translations through nucleotide complementarity [1]. The cleavage site primarily locates to the 10th miRNA nucleotide from the 5'-end. To verify that miRNAs can regulate their target mRNA expression in *P. densata *by cleavage, we carried out a modified RLM-5' RACE experiment, using total RNAs extracted from seedlings (see Methods) [42].

**Figure 5:**
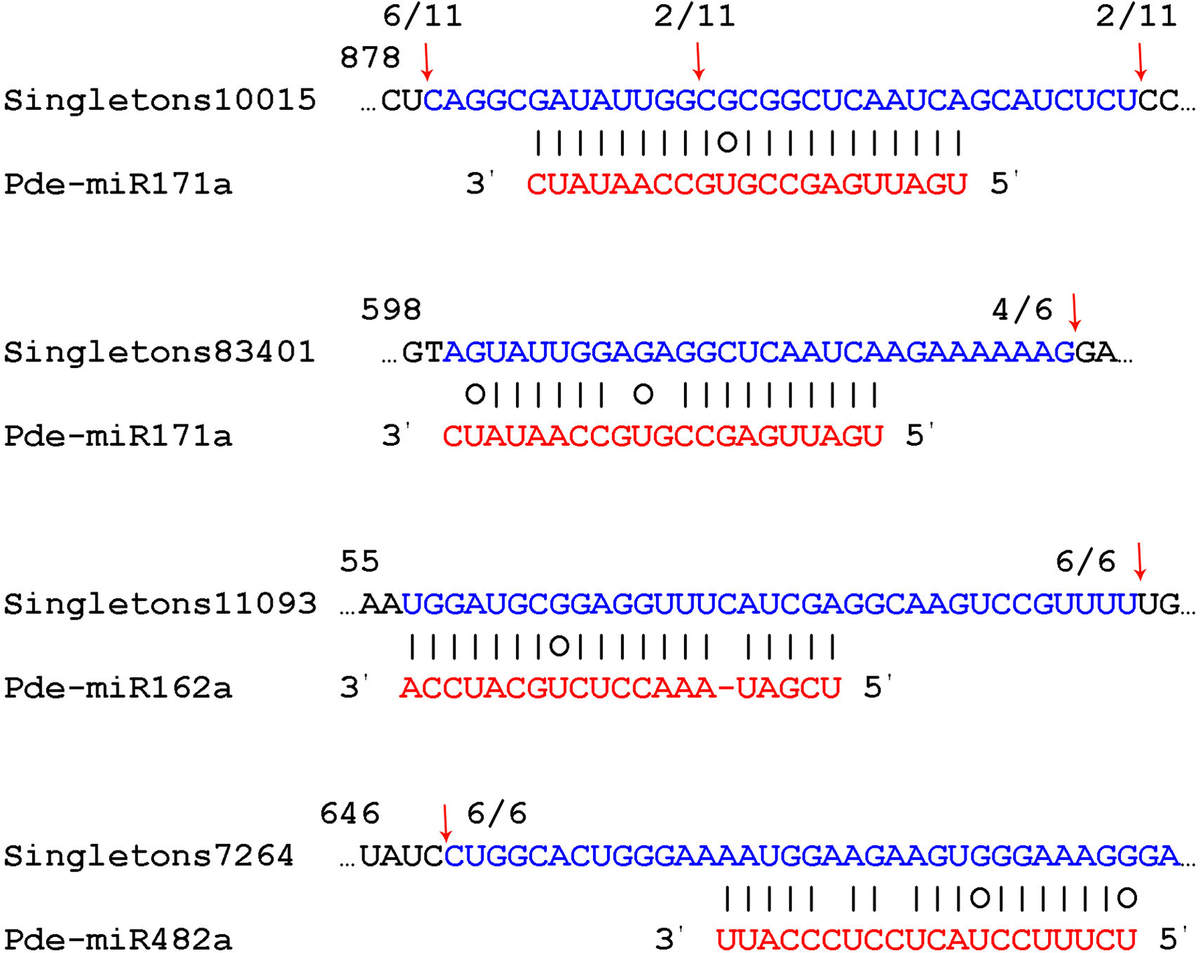
**Detection of miRNA-mediated mRNA cleavage**. Partial mRNA sequences from target genes were aligned with corresponding miRNAs. Each top strand (blue) represents a miRNA complementary site in the target mRNA and each bottom strand (red) represents the miRNA. G:U wobble pairing (circles) and Watson-Crick pairing (vertical dashes) are indicated. Red arrows indicate the 5' termini of the degraded mRNA fragments isolated from *Pinus densata*, which is identified from cloned 5' RACE products, with the frequency of clones shown.

In the present study, we successfully detected the cleavage sites in four predicted target genes of *P. densata *(Figure 5). Singletons10015, singletons83401, singletons11093 and singletons7264 were confirmed to be targets of pde-miR171a, pde-miR162a and pde-miR482a, respectively. We observed a shorter or longer cleaved sequence for three putative targets, singletons83401, singletons11093 and singletons7264, after 5' RACE analysis. It could be attributed to secondary siRNA in the 21-nucleotide register with the cleavage site for miRNAs, as reported by Ronemus and De Paola [43,44]. Singletons10015 is similar to plant proteins coded by GRAS family transcription factor protein, while singletons83401, singletons11093 and singletons7264 coded for proteins homologous to actin binding protein, nodal modulator 1-like protein and histone deacetylase, respectively.

## Discussion

### Conserved miRNAs in *P. densata*

Previous studies have identified thousands of miRNAs in angiosperms and some of them have been well-characterized [45,46]. Few miRNAs from gymnosperms have been reported to date [30-34]. *P. densata *is an ecologically important conifer in East Asia. It is an ideal model plant to study homoploid hybrid speciation. However, only 399 EST sequences are available in the public databases to date. No miRNAs from *P. densata *has been reported in previous research. In order to obtain global mRNAs from *P. densata*, we recently sequenced an mRNA library constructed from total RNAs of needles using the Illumina high-throughput sequencing technology. Since pri-miRNA sequences also possess poly(A) tails and thus can be isolated and sequenced as mRNAs, it allows us to identify candidate pri-miRNA sequences from the *P. densata *mRNA transcriptome database by bioinformatics tools [1]. In this study, a total of 34 conserved miRNAs belonging to 25 miRNA families were identified from *P. densata*. The primary and/or precursor sequences of 29 miRNAs were further confirmed by subcloning and sequencing. In addition, 19 novel mature miRNAs belonging to 14 conserved miRNA families were found through the analysis of their precursor sequences. The 34 pre-miRNA sequences could form hairpin structures as predicted by MFOLD. They had an average MFEI value of 0.92 (Table 1). Nineteen mature miRNAs (58%) had a 5' terminal uridine residual, which is a characteristic feature of typical miRNAs. These results indicate that the 34 candidate miRNAs may be canonical. The miRNAs identified in the present study can definitely provide useful information for further study on their regulative functions and biogenesis of them.

Among the 21 miRNA families conserved between dicots and monocots, 8 of which were identified in *P. densata*. It includes pde-MIR159, pde-MIR162, pde-MIR166, pde-MIR169, pde-MIR171, pde-MIR390, pde-MIR396 and pde-MIR399. It indicates that the ancient miRNA regulatory system is well-developed in the common ancestors of gymnosperms and angiosperms [47]. Compared with *P. taeda *and *P. abies *miRNA families listed in miRBase version 16, five new families (pde-MIR162, pde-MIR169, pde-MIR399, pde-MIR1448 and pde-MIR2118) were only found in *P. densata *[30,34]. Three *P. taeda *miRNA families, including pta-MIR319, pta-MIR398 and pta-MIR408, were not found in *P. densata*. It could be due to the simplicity of tissue used for Illumina sequencing.

We also found a few contigs highly homologous to 12 conserved miRNAs. They were excluded for further analysis because they were not able to form canonical hairpin structures. With whole genome sequences and larger EST databases, more miRNAs will be identified from *P. densata*.

### Expression profiles of miRNAs

In the present study, we found the expression levels of 7 conserved miRNAs were more than 2-fold higher in needles than in stems, using real time RT-PCR. It suggests that these conserved miRNAs may play specific roles in a variety of physiological processes in needles. Previous publications have shown that many miRNAs have important functions in the establishment of leaf polarity and virus-induced leaf curling. Zma-miR166a is an important miRNA known for establishing the adaxial/abaxial (dorsoventral) leaf polarity by repressing the expression of class III homeodomain leucine zipper (HD-ZIPIII) transcription factors in maize [48]. The accumulation of miR159 was observed to increase with the days post inoculation (dpi) of *tomato leaf curl New Delhi virus *(ToLCNDV) agroinfection in tomato cv Pusa Ruby [49]. In our study, pde-miR159 and pde-miR166a were found highly expressed in *P. densata *needles. Meanwhile, Singletons59617, coding for a protein highly homologous to Class III HD-Zip protein HDZ33, was predicted to be the target of pde-miR166a. These results suggest that the two miRNAs may also involve in needle polarity establishment and antiviral reaction in *P. densata*.

### MiRNA targets and their putative functions

To define and elucidate the putative functions for a miRNA in plant, a necessary step is to predict and validate its target mRNAs. Currently, the most efficient tool available for this purpose is the bioinformatics approach, which is based on perfect or near perfectly complementarity between miRNAs and their targets. In this study, we predicted miRNA targets in *P. densata *using an online miRNA target search program, psRNATarget [41]. Our results showed that *P. densata *miRNA targets encoded not only indispensable transcription factors, but also non-transcriptional factor proteins involving in diverse physiological processes. For example, HD-ZIP and GRAS family transcription factors, which are important to root and nodule development in *Medicago truncatula *and nutrient homeostasis in maize, were predicted to be targets of pde-MIR166 and pde-MIR171, respectively [50,51]. Non-transcriptional factor proteins, such as DCL1 and CC-NBS-LRR resistance-like protein, were predicted to be targets of pde-MIR162 and pde-MIR2118, respectively [52]. DCL1 is one of the essential components involving in the miRNA biogenesis. The prediction of DCL1 to be the target of pde-miR162 implies that the miRNA biogenesis process is self-regulated in *P. densata*.

To validate the miRNA targets in *P densata*, we performed a modified RLM-5' RACE experiment. In the present study, 4 miRNA targets, including singletons10015, singletons83401, singletons11093 and singletons7264 were confirmed to be targets of pde-miR171a, pde-miR162a and pde-miR482a, respectively. These results suggest that miRNAs can regulate the expression of their targets by cleavage in *P. densata*.

## Conclusions

In summary, we performed a transcriptome-wide identification and characterization of miRNAs from *P. densata*. A total of 34 conserved miRNAs comprising 25 miRNA families were identified. The primary and/or precursor sequences of 29 miRNAs were confirmed by subcloning and sequencing. Analysis of miRNA precursors revealed 19 pre-miRNA sequences harboring novel mature miRNAs belonging to 14 conserved miRNA families. Seventeen of the novel miRNAs were validated by sequencing. Using real-time quantitative RT-PCR, we found that the expression levels of 7 *P. densata *miRNAs were more than 2-fold higher in needles than in stems. Utilizing a Web-based program psRNATarget, 72 mRNAs were predicted to be targets of 25 miRNA families. Four mRNAs were experimentally validated to be targets of 3 *P. densata *miRNAs by RLM-5' RACE. These results suggest that regulative miRNAs exist in ecologically important conifer, *P. densata*, and may play key roles in *P. densata *development, growth and response to environmental stress.

## Methods

### Plant material

*P. densata *needles were harvested from a mature *Pinus densata *tree in Linzhi city, Tibet, China and stored at -80°C until use. *P. densata *seedlings were grown under standard greenhouse conditions.

### Total RNA isolation and Illumina sequencing

Using RNAiso-mate for plant tissue and RNAiso plus (Takara, Dalian, Liaoning, China), total RNAs were purified from needles and treated with RNase-free DNase I for 30 min at 37°C (Promega) to remove residual DNA. Total RNA of needles was used for Illumina sequencing of mRNA transcriptome, which was performed at Beijing Genomics Institute (BGI), Shenzhen, China. After removing reads containing only 3' sequencing adapters and reads of low quality (reads containing Ns > 5), transcriptome de novo assembly was carried out with short read assembling program - SOAPdenovo [53].

### Identification of *P. densata *miRNAs

In order to find conserved miRNAs, we aligned 3,968,794 unique sequences from the mRNA transcriptome database of *P. densata *against the mature and precursor sequences of known plant miRNAs deposited in miRBase version 16 http://www.mirbase.org/, using local BLASTN and MIREAP programs http://sourceforge.net/projects/mireap/[36,54]. To discover novel miRNAs, 166 and 191 small RNAs from *Pinus taeda *and *Picea abies *were downloaded and used in the analysis as described above [30,34]. Sequences with an *E*-value of lower than -2 or a score > 32 were processed for further analysis, allowing for a maximum of 2 nt mismatches. Overlapping contig sequences were used to form longer sequences according to their alignments to known precursor sequences in the miRBase. MFOLD was employed to predict hairpin structures with default parameters http://mfold.bioinfo.rpi.edu/cgi-bin/rna-form1.cgi[55]. Sequences were considered as miRNA precursor sequences if they met the following criteria: the RNA sequence could form an appropriate stem-loop structure, with a mature miRNA sitting in one arm of the hairpin structure; mature miRNAs had less than 6 mismatches with the opposite miRNA* sequences in the other arm; the predicted secondary structure had a minimal folding free energy of less than or equal to -15 kcal/mol, a minimal folding free energy index of more than 0.5, and a 30-70% A + U content [26].

### Subcloning and sequencing of pri- and pre-miRNA sequences

Total RNA was isolated from 2-month-old seedling stems as described above. cDNAs were synthesized from 2 μg of purified total RNA in 25-μl reactions, containing 200 U M-MLV reverse transcriptase (Promega, Madison, WI, USA) and 1 μg random nonamer, according to the manufacturer's protocol. The housekeeping gene *Actin *was used as a positive control. Thirty-eight pairs of primers for *P. densata *primary or precursor sequences were designed (Additional file 7). PCR amplifications were carried out, using the following thermal cycling conditions: 94°C for 3 min, 35 cycles at 94°C for 30 s, 55°C or 60°C for 15 s and 72°C for 50 s. Amplification fragments were separated on a 2% agarose gel with ethidium bromide (EtBr) staining. Gel-purified PCR fragments were subcloned into pGEM-T Easy Vector (Promega) and sequenced.

### Quantitative real-time PCR

Total RNAs were purified from needles and stems of 2-month-old seedlings as described above. Reverse transcription was carried out using 1 μg of total RNA and the NCode miRNA First-Strand cDNA Synthesize Kit (MIRC-50; Invitrogen) following the manufacturer's recommendations. The resulting cDNA was diluted 10 times with sterile water. Quantitative real-time PCR was performed in triplicate reactions using the MX3000P detection system (Stratagene, La Jolla, CA, USA). Ten forward primers were designed based on mature miRNA sequences. If the T_m _of a mature miRNA was < 60°C, it would be adjusted by adding Gs or Cs to the 5' end and/or As to the 3' end of the miRNA sequence (Additional file 7) [34]. A 20-bp segment at the 3' end of the 5 s rRNA gene was amplified as an endogenous control to normalize template amounts. Since many miRNA paralogs differ by only 1 nucleotide, we adopted stringent annealing conditions and set the annealing temperature to 65°C for quantitative real-time RT-PCR reactions [56]. Quantitative PCR reactions were conducted in 20 μl volumes containing 2 μl diluted cDNA, 300 nM of each primer, and 10 μl of the Thunderbird SYBR Green PCR Master Mix (Toyobo, Tokyo, Japan) with the following cycling conditions: 95°C for 1 min, 40 cycles at 95°C for 15 s, 65°C for 15 s, and 72°C for 5 s. After amplification, a thermal denaturing cycle at 95°C for 15 s, 65°C for 15 s, and 95°C for 15 s was carried out to determine the dissociation curves and verify the specificity of the amplifications. All expression levels were normalized to the arithmetic mean of the selected 5 S ribosomal RNA gene. Amplification results were analyzed using a comparative C_t _method, which uses a formula, 2^-ΔΔCt^, to achieve results for relative quantification. C_t _represents the threshold cycle. The expression level in stems was arbitrarily set to 1 [30].

### Prediction of miRNA targets

To identify putative targets of *P. densata *miRNAs, we used 38 mature miRNAs as custom miRNAs and 3,968,794 sequences in the *P. densata *mRNA database as custom mRNAs to search for complementary hits using the psRNATarget program http://bioinfo3.noble.org/psRNATarget/ with default parameters. The score system was applied according to Zhang [41]. Sequences with a penalizing score ≤ 3 were chosen as putative targets. We further performed BLASTX searches against the NCBI database to identify putative gene homologs. Similarities with an *E*-value of less than e^-10 ^were considered a hit.

### Validation of miRNA-mediated cleavage of mRNA

To identify cleavage sites in the target mRNAs, a modified RNA ligase-mediated rapid amplification of cDNA ends (RLM-RACE) was performed using a 5' RACE kit (Takara) [42]. Total RNA was isolated from seedlings as described above. An RNA Oligo adapter was directly ligated to the purified total RNAs (2000 ng) without calf intestinal phosphatase and tobacco acid pyrophosphatase treatment. Twenty pairs of nesting and nested gene-specific primers were designed and applied for PCR amplifications. Six DNA bands with expected sizes were gel purified and cloned into pGEM-T Easy Vector for sequencing. Four of the sequenced DNA bands were identified to be miRNA-guided cleavage products.

## Abbreviations

AGO: Argonaute; DCL: Dicer-like; GO: Gene Ontology; MFEI: Minimal folding free energy index; miRNA: MicroRNA; pre-miRNA: Precursor miRNA; pri-miRNA: Primary miRNA; RISC: RNA-induced silencing complex; RT: Reverse transcription.

## Authors' contributions

LW designed and carried out the study and drafted the manuscript. HZ and LZ participated in the bioinformatics analysis. SL, ZQ and YZ helped to draft the manuscript. QZ and JL conceived of the study, participated in its design and coordination and helped to draft the manuscript. All authors read and approved the final manuscript.

## Supplementary Material

Additional file 1 **Precursor sequences of *P. densata *conserved miRNAs**. Click here for file

Additional file 2 **Primary sequences of *P. densata *conserved miRNAs**. Click here for file

Additional file 3 **Predicted hairpin structures of *P. densata *miRNA precursors**. The hairpin structures of *P. densata *miRNA precursors were predicted by MFOLD. Mature miRNAs were marked in yellow. Click here for file

Additional file 4 **Predicted stem-loop structures of precursors containing *P. densata *microRNA sequences (red and blue)**. Click here for file

Additional file 5 **Conserved mature miRNAs without hairpin structure in *P. densata***. Click here for file

Additional file 6 **Predicted targets of *P. densata *miRNAs and their putative functions**. Click here for file

Additional file 7 **Primers used for amplifying *P. densata *miRNAs and their targets**. Click here for file
