# Supplementary material for: Transcriptome-wide identification and characterization of miRNAs from Pinus densata
Source: BMC Genomics. 2012 Apr 6;13:132. doi: 10.1186/1471-2164-13-132 (PMC3347991; doi:10.1186/1471-2164-13-132)
Supplement: Additional file 1 — Precursor sequences of P. densata conserved miRNAs. [file 1471-2164-13-132-S1.DOC]

**Additional file 1 Precursor sequences of *P. densata* conserved miRNAs.**

| **miRNA name** | **Precursor sequence (5'-3')** | **Length (nt)** |
| --- | --- | --- |
| pde-miR159a | UAAGCGGUAGAGCUCCUUUUGUACCAAUCAAGGACUGUGCAAAAAUGAUCCGACUGCCGAUUUAUGCAUCCUUCUGCCCUGCGAGUGUUCGUUGUUUCAAGUCCGAAUAGAUUGCAGGUUAGUGGUUUGCAGAAGACCGAUUUCAUUGCAGGUUAGUGGUGUGCAUAAGGCGGGAGUUGUAUUGAUUUUGUGUUGUCCUCGUUUGGUUUGAAGGGAGCUCUACUGGGC | 228 |
| pde-miR162a | GCGAUUUCAGACGCUUGGCAGCCUGGAUGCAAAGGGUUUACCGACCAUGUCCACGGGCCGCUGGGUCUCGGUCGAUAAACCUCUGCAUCCAGAUUGUUUGGAAUCUGUUGC | 111 |
| pde-miR166a | GGGGAAUGUUGUCUGGCUCGAGGCCAUCCUGAAAAUAGUCCACCCCCGGUUCUUUUGGGGUUUUUUUGGAUGCCGUCGGACCAGGCUUCAUUCC | 94 |
| pde-miR166b | GGGGAAUGUUGUCUGGCUCGAGGUCACUCAGAUUUGAUGACGAUGAUUCUUUGAGCUGAGUGACGUCGGACCAGGCUUCAUUCC | 84 |
| pde-miR169a | ACUAUUUAUUCAGCCAAGGAUGACUUGCCUAGAUCUUGUUACCAGUAUCGGCUAGUUAUGCUGAUAUCGAUUUCAGUCACUAGGUGAGUCGUCCUUGGCUAAA | 103 |
| pde-miR171a | GAAAGAAUGUGAUGUUGGCUAGGCUCAAUCGGAUUGUAACGCCCACGGAAUUUGGUCUUGUGAUCUGAUUGAGCCGUGCCAAUAUCACAUUCUAAC | 96 |
| pde-miR390 | UAAUGGUAUAAAGAAAUUAUGAAGCCCAGGAUGGAUAGCGCCAGCCCCACUUGAAAUUUGCAGUGGGCGCUAUCCCUCCUGAGCUUUGUAAUUCC | 95 |
| pde-miR396a | UUUUCCCACGGCUUUCUUGAACUUCUCAUUCGGAACUGUUAUCAGUGCGUCCGGCCGAUGCCCACAGAACUCAUCAUGAAGUUCAAGAAAGCCGUGGAAAAAUAUAA | 107 |
| pde-miR482a | UGAGAAGUGAAGGGAUGUGUUUUGUGGAUGGGAGUCUUGAGGAGUGGGAGCAUAGGAUAAGGCUGCUUCAUAUCACCAGUCUUUCCUACUCCUCCCAUUCCUAUUGCCUUCACCACACAUCCCUUCCCAA | 130 |
| pde-miR482b | AUGUGGAUGGAAGUCUUGAGGAGUGGGAGGGUAGGAGAAGGCUCUGUGGUGAGGUUUCAGUCAUAAUCUCAUCAGUCUUCCCUAUUCCUCCCAUUCCUAUUGCCUCCCUUCACAAUGUAAC | 121 |
| pde-miR482c | AAGGCCAAUGGCUUGCGAGGGUAGGAAAAGCUCAGUGUGAUGAUAUAUUUCUCGCUCACUGAUCUGCAGUUUUUCCCACUCCUCCCAAGCCCAUGGCC | 98 |
| pde-miR482d | GUCAUGGGGUCUUUAGGCUUUGGAGGAUUUGGAAAGGCUUAGUCAUUCUUUUUACCGAGGAUAUUCGAUUUUCUAAGAACCUUUCCAACGCCUCCCAUGCCUAUAGUCCUCAUUGUACAUGUCACCCGAUCGCAG | 135 |
| pde-miR783 | UUCUUUUGAGGGAAGGGAGCUGGCGUGCAUUUCUUUUCAGAUAUUCAUUGCGUUCGCCAAGAUUCUUUGCUGGUUCAUUUUCCCUGAGAUGAA | 93 |
| pde-miR946a | CAGAGUGUAUAGUUGUGGAUACAGAAGGGUUAGUAAACGGUAGAAAACCCAUUUAUUGUAUAUCAUUGGCUGUUCGAGAUUAAGCACCUGUAAUUCGAUGGCCAUACUCAUUAAAUGGGUUGCCUGUUUGCUCUGUACCAGCCCUUCUCCUAUCCACAAUUGUACUAGUUUG | 172 |
| pde-miR947 | ACGCCUAAGGCGCAGCAGCAGAUUCUGAUAGAAGACUCAGGCAAAGCAUUUGUUGUUGGCAGGUUGAAUGCUUUCGGCAGAACCACCAAUAACAAGGGGGGGCGCCUGGUGUCUUGCAUCGGAAUCUGUUACUGUUUCCUAGGCGU | 146 |
| pde-miR949a | CAGAGCUUCUCUAGGAAUCAAAUGUGUCUUCCUCUUGAACGCCUUUCACGCACAGGAAGGCCCAUUGGAUUCCCGGGAGAAGCUCUCC | 88 |
| pde-miR949b | AGAGCCUCUCCGGGAAUCCAAUGCGCCUUCCUCUUGAACGCCUUUCAACGCGCAGACUGUGCGUGAAAACGCUUUUAGGAGGAAGGUGUAUUGAUUUCCGGGGAAGCCCU | 110 |
| pde-miR950a | GAAGGUGAUCUUUACAUCUGGUCCACGGUGGUUUAUUGUUCAUCGUUUGUUUAUGAUAAACCAUCGUGCCGCAGAUGAUAAGAGCACCUUC | 91 |
| pde-miR951 | GAAGCGAUGGUGUUCUUGACGUCUGGACCACGUGGGUUUGCUUUACGUUGGGCAUGAAUAAACAAUCAUCUACCGCGGUUCAGUCAUCAAGAACACCUUUGCUUUCAUUUACAUG | 115 |
| pde-miR952a | GCGAGCUAUCGAAGGAGAGAACCAGUGGCGUAUUGAACAGAGCAUGCCAUUGGUGGAGUAAGUACGUCAAGGCACGAAACAGAAUUAAUUUUGAUUAACGUUUUAUUAACCUUCAACUCUGCAUUGGCCUAUGGCAGUUCCUCAAGGUCACAUCGAUGGACAACUCUGCAUUGGCCUAUGGCAGUUCCUCAAGGUCACAUCGAUGGACUGGGGCGUGAUACAAGGCGUAGUCCGCUGGUUUCUGCCACCGUUAGGACUCUCCCCAACCUAUUCUCAGCUGCAACAUUUACAACUGCAACAUUCACAAGUUAUUUACAAUGUUGCAGCCGAGAAUAAAAUGGGAAGACUCCAAAGCGUCGCAACAACCAGCUUUGUAUCAGCCCCCCAACUGGUGUGAGUUUGGGGAACUGCCACAUGCCAAGCCAGACUUGCAUGCUAAUAAAACAUUAUUCGAAAUUGCUUCUAUUUUGUUUGUUGACAUACUUCCUCCGCCAAUGACACGCUCAGUUCAAUUUGCUGAUGGUUC | 526 |
| pde-miR952b | GCGAGCUAUCGAAGGAGAGAACCAGUGGCGUAUUGAACAGAGCAUGCCAUUGGUGGAGUAAGUACGUCCAGGGACGAAACAGAAUUAAUUUUUUUGAUUAACAUUUUAUUAACCUCCAACUCUGCCUUGGCCUAUUGCAGUUCCUGAAGGUCACAUCGACUGGGGCGUGAUACAAGGCGUAGUCGGCUGGCUGCUGCCACCGUUGGGACUCUCCCAACCUAUUCUCAUCUACAACGUUAAAAAGUUACUUACAUUGUUGCAGCCGAGAAUAAAAUGGGAAGACUCCAAAGGGUCGCACCAACCAGCCAAUUGCGCUUUGUAUCAGCGCGCCAACUGGUGUGAGCUUUGGGAAUUGCCAUACGCCAAGCCAGACUUGCAUGCUAGUAAAACAUUAUUCGAAAUUACUUCUAUUUUGUUUGUUGACAUACUUCCUCCGCCAAUGACACGCUCAGUUCAAUUUGCUGAUGGUUC | 472 |
| Pde-miR952c | GCGAGCUAUCGAAGGAGAGAACCAGUGGCGUAUUGAACAGAACAUGCCAUUGGUGGAGUAAGUACGUCAAGGCACGAAACAGAAUUAAUUUUGAUUAACGUUUUAUUAACCUUCAACUCUGCAUUGGCCUAUGGCAGUUCCUCAAGGUCACAUCGAUGGACUGGGGGCGUGAUACAAGGCGUAGUCCGCUGGUUUCUGCCACCGUUGGGACUCUCCCCAACCUAUUCUCAGCUGCAACAUUCACAAGUUAUUUACAAUGUUGCAUCCGAGAAUAAAAUGGGAAGACUGCAAAGCGUCGCAACAACCAGCUUUGUAUCAGCGCCCCAACUGGUGUGAGUUUGGGGAACUGCCACAUGCCAAGCCAGACUUGCAUGCUAGUAAAACAUUAUUCGAAAUUACUUCUAUUUUGUUUGUUGACAUACUUCCUCCGCCAAUGACACGCUCAGUUCAAUUUGCUGAUGGUUC | 465 |
| pde-miR1310 | AUUAGAGGCAUCGGGGGCGUAACGCCCUCGACCUAUUCUCAAACUUUAAAUAGGUAAGAGGGUGCGGCUGCUCCAUUGAGCCG | 83 |
| pde-miR1311 | GUAGGAACAGGCGGACUGGCAUAACUCCGCCCAUAUUUUACUGUCAGAGAUGGUCAGAGUUUUGCCAGUUCCGCCCAUUCCUACUG | 86 |
| pde-miR1312a | UCCCCCAAAACAUCCAUAUCGCUAUAUCUCUCCUAAUUAGGUUGAAUCGAAACAGCCAACACUUUCAGUUGAUUCAAAUAUUAGUUUGUUUCUGCAUAAUUUGGAGAGAAAAUGGCGACAUAGAUGUUUUCGGCGAU | 137 |
| pde-miR1313 | UCGUGAUGGUAUUCUACCACUGAAAUUAUUGUUCGAAAUAACACCUGCAAUUCAUUGAUGUAUACCACCGUGGUAUGGCAUCCUUCAGCGUUUCGCCAAACUAACCUAGUUCCAGGGUUGCAACACAUGGCAUGGUGGAGGAUCUAUUGGGAAAUAACCAAGUUGCAUGAGUGUAUUUAUCCAACAAUAAUUUCAGUGGAAGACUUCCAUCGAUCUGGAAGACUCAU | 227 |
| pde-miR1314a | GUCAUGUUGGUUAGAUGAAGGAAUUCUUCAACAUUAGAGGCCGAUGUGGAGUUAUGCUCUUGACCGGCCUCGAAUGUUAGGAGAAUGUUUUCCUCUACCUAAGCA | 105 |
| pde-miR1448 | GUCAUGGGGUCUUUAGGCUUUGGAGGAUUUGGAAAGGCUUAGUCAUUCUUUUUACCGAGGAUAUUCGAUUUUCUAAGAACCUUUCCAACGCCUCCCAUGCCUAUAGUCCUCAUUGUACAUGUCACCCGAUCGCAG | 135 |
| pde-miR2118a | GUCAUGGGGUCUUUAGGCUUUGGAGGAUUUGGAAAGGCUUAGUCAUUCUUUUUACCGAGGAUAUUCGAUUUUCUAAGAACCUUUCCAACGCCUCCCAUGCCUAUAGUCCUCAUUGUACAUGUCACCCGAUCGCAG | 135 |
| pde-miR2118b | GAGGAGUGGGAGCGUAGGAGAAGGCUCUGUGGUGAGGUCUCAGUCAUAAUCUCAUCAGUCUUCCCUAUUCCUCCCAUUCCUAUUG | 85 |
| pde-miR3701 | CGCCACAGAUGAAGGGUUGGAGUUGUUCGGUAGAAGAGUUUCAUCUGUUGUAGGAUAUGGAGGAUUUUCCAAAAUAUUUGCCAUCAAAUUAAAAGACUAUAGUGGUUUCAUUGCAAUUUGUUUGGCACUAUCCUCUAUAUCCUUCUGCCCUAAAAUUCUUCUCCUGAACAAUGCCCACCCUUCAUCUCUGGGCA | 194 |
| pde-miR3704a | AAGGUCAUGGGUCUCGGUGGAGUUGGGAAGACUGUUACAGUGCAUAGAUGUCAUCAGAUCAUAAAUUACUGCGUAGGUAUUUCCAACCCACAGAGACCUGAUGGCCUU | 108 |
| pde-miR3704b | AAGGUCAUGGGUCUCGAUGGAGUUGGGAAGACUGUUACAGUGCAUAGAUGUCAUCAGAUCAUAAAUUACUGCGUAGGUAUUUCCAACCCACAGAGACCUGAUGGCCUU | 108 |
| pde-miR3712 | GAGAUGUGAUCAAGAUCAGACUCCCAAAUCCAACAGCAUUUGAGGUCUUUGCUCGAUUAAUAUCAUCCA | 69 |
