# Supplementary material for: Transcriptome-wide identification and characterization of miRNAs from Pinus densata
Source: BMC Genomics. 2012 Apr 6;13:132. doi: 10.1186/1471-2164-13-132 (PMC3347991; doi:10.1186/1471-2164-13-132)
Supplement: Additional file 2 — Primary sequences of P. densata conserved miRNAs. [file 1471-2164-13-132-S2.DOC]

**Additional file 2 Primary sequences of *P. densata* conserved miRNAs.**

| **miRNA name** | **Primary sequence (5'-3')** | **Length (nt)** |
| --- | --- | --- |
| pde-miR159a | UUUUUUUAGGUAUGUUCAUAUGGUUUAAAGUGGAAGGUCUGGUAAGCGGUAGAGCUCCUUUUGUACCAAUCAAGGACUGUGCAAAAAUGAUCCGACUGCCGAUUUAUGCAUCCUUCUGCCCUGCGAGUGUUCGUUGUUUCAAGUCCGAAUAGAUUGCAGGUUAGUGGUUUGCAGAAGACCGAUUUCAUUGCAGGUUAGUGGUGUGCAUAAGGCGGGAGUUGUAUUGAUUUUGUGUUGUCCUCGUUUGGUUUGAAGGGAGCUCUACUGGGCGAC | 273 |
| pde-miR162a | CGUGAGGGAGAGGACAUGCAGUCUAUUUAGGUAUCCGAGUGGGCGCAAACGGAAUCGAUCAGUGGCGGGACGACCUUUGGGCGGGCGAUUUCAGACGCUUGGCAGCCUGGAUGCAAAGGGUUUACCGACCAUGUCCACGGGCCGCUGGGUCUCGGUCGAUAAACCUCUGCAUCCAGAUUGUUUGGAAUCUGUUGCUUCUCUCCCUUUGUGAUGAUGACAACUCUUCCCGUGCACGCAGGAAAUUGCAUAUUCUUCUGUUAGUGUUUUGUUGCCCGGGGAGCUUUUUGGCCGGUGGCCUUGGGGUUUCCGUCUUUAUUUUUAUUUUUAGUGGGGCGUAUUUUAAUCUUGUUUCAUUUUUUAAAUAUG | 366 |
| pde-miR166a | GAGGACGAGGAGGGCAAGAAGUAGAAGAAGCAGCCAUUGAAGUUGAAGAAGAAAUGGGUUUGAGGGGAAUGUUGUCUGGCUCGAGGCCAUCCUGAAAAUAGUCCACCCCCGGUUCUUUUGGGGUUUUUUUGGAUGCCGUCGGACCAGGCUUCAUUCC | 157 |
| pde-miR166b | GACAUUGAAGGUGUCCUCGUUGUGAAGGAUUUGCUGGUGCUUCUUGGUUUACGAUUGCGGGGAAUGUUGUCUGGCUCGAGGUCACUCAGAUUUGAUGACGAUGAUUCUUUGAGCUGAGUGACGUCGGACCAGGCUUCAUUCC | 142 |
| pde-miR169a | AAAAAAAAUUCAUUUGAAAUGGCCAAAAAGCCAUCUUUCUGCUGCAACUAUGGGUUCGUUUCUUCAGGUUUGGAUAGCAUUUGGAAUUUGCUUGUCCCAUAAAUUUCAUAUGGGAUUCUCAAUGUAAUAUUAUGCGAGCUUCGUGGAUGGAACAGCGUUUGGUGUGUGUUCAUCUGUGAGCUUGCAUGCAGAGGCAGAACUAUUUAUUCAGCCAAGGAUGACUUGCCUAGAUCUUGUUACCAGUAUCGGCUAGUUAUGCUGAUAUCGAUUUCAGUCACUAGGUGAGUCGUCCUUGGCUAAAUAUUUUUAUCUGCGUCUUAUGCUAGACCUCAGAUGAGGAAAAGUUUAGAUCAGCUAGAUUGUAUGUAUCAGUUCUACUUUCUUAUUGAAUUUGAACAAGCGAUACUUCAGCUGUGUGUUUUGUAUUUCAGGGAAGGAAAUGCAAAUUCUCUGGGCUUCCAC | 462 |
| pde-miR171a | GCAGAUGGUACAGUGUUGUAAACGCGGGAGGAAAGGGCGGUUAGGGUCUGCAGCAGCUGAAAGAAAGAAUGUGAUGUUGGCUAGGCUCAAUCGGAUUGUAACGCCCACGGAAUUUGGUCUUGUGAUCUGAUUGAGCCGUGCCAAUAUCACAUUCUAACAUUCAUCCACUCCAUUCCAUUCCAUU | 184 |
| pde-miR390 | UAAUGGUAUAAAGAAAUUAUGAAGCCCAGGAUGGAUAGCGCCAGCCCCACUUGAAAUUUGCAGUGGGCGCUAUCCCUCCUGAGCUUUGUAAUUCC | 95 |
| pde-miR396a | UUUUCCCACGGCUUUCUUGAACUUCUCAUUCGGAACUGUUAUCAGUGCGUCCGGCCGAUGCCCACAGAACUCAUCAUGAAGUUCAAGAAAGCCGUGGAAAAAUAUAACAGGAUUCUCGGCCAGAGUACGUCCUUAUUUUCUAACUGCGUAUACAUGCUGCUAAUAACUGGAUCGCAUCAUUACUAAUAAUAAGUUUUCCCGAAUCCCAAU | 210 |
| pde-miR482a | GGCGAGAAGGCCUUAUUGUUUUGUUGGUUGUGAGAAGUGAAGGGAUGUGUUUUGUGGAUGGGAGUCUUGAGGAGUGGGAGCAUAGGAUAAGGCUGAUUCAUAUCACCAGUCUUUCCUACUCCUCCCAUUCCUAUUGCCUUCACCACACAUCCCUUCCCAACGCCAUACAAUAUGUGAAGAUGAAUAUGGAAAACCAAUCGUCGUGCUCUGUUCUGUGGGGUUUUACCGGGAUGGCAUGUAGUUUUAAGGCCAUUUGGUCUCGGUAAGUUGGAAAAACUUGGCAGUAAUUCAUGAUCUUAUCUGAUCCGGUAUGAUAUAUUAACUGUUGCAGUUUUCCAAACCCGCCGAGACCCAUUGGCCUUCACUACACAUCCCUA | 377 |
| pde-miR482b | UUUGUUGAUUUUGAGAAGGGAUCUGUCAUGUGGAUGGAAGUCUUGAGGAGUGGGAGGGUAGGAGAAGGCUCUGUGGUGAGGUUUCAGUCAUAAUCUCAUCAGUCUUCCCUAUUCCUCCCAUUCCUAUUGCCUCCCUUCACAAUGUAACACAGUACACAGCCUGUUAAAGAAACGAAUAUGGCUAAAUAUCAUUCUGUUUUGUGGGUAUUAUGGGGAUGUCCUGUAGUUUUAAGGCCAUCAGGUCUCUGUGGGUUGGAAAUACCUACGCAGUAAUUUAUGAUCUGAUGACAUCUAUGCACUGUAACAGUCUUCCCAACUCCACCGAAACCCAUGACCUUCUUCACUACACAUCCCUUUUGUGCCCGCUCUAUUAACUCUGGCGCUGUUACUCUCCAGAUCUGCAAACAUGGCACAGGGGCAUGGCUGCAACAUACAGUUGAUUGUAAGCCUGUCCCUGCUUUAUGUCGUGAAUUUAUUUAUUUUUGCAGGGGACAUGGAGCUUAUUAUACUGUGACCAAGAUCUGGAUUUGGGUGUAUGGCUUGGAUGUUAGAACAAGAAUUAGUUCUGGAUUUGGGUGUAUGGCUUGGAUGUUAGAACCAGCAUUAGUUUUGUGUUUCCUUUCUUAGAGUACCAUGUAAUAAUUGUAGAGAAGUCAAUUUGAUUGUAUAAAUUGUUUGAUAGUAGGGAUGCCUGUUUAGAGAGGAUUAUGGUAUGUAAGAACCUUUAUUUUAAGGUCUCAACCUAUCAUAUUAAGCUGUUGAAAUUAGUAAUAACAGAUCUCAAGUUCAGGCAAAUAAAAAUGUCUCCG | 809 |
| pde-miR482c | GAAAGGCCAAUGGCUUGCGAGGGUAGGAAAAGCUCAGUGUGAUGAUAUAUUUCUCGCUCACUGAUCUGCAGUUUUUCCCACUCCUCCCAAGCCCAUGGCC | 100 |
| pde-miR482d | GUCAUGGGGUCUUUAGGCUUUGGAGGAUUUGGAAAGGCUUAGUCAUUCUUUUUACCGAGGAUAUUCGAUUUUCUAAGAACCUUUCCAACGCCUCCCAUGCCUAUAGUCCUCAUUGUACAUGUCACCCGAUCGCAG | 135 |
| pde-miR783 | AGGGACCAACAGGAUUGAUGCGCUACCAUUCGGCGCCCAGCUCAUUUUUUUCAAGUUUAGGAGAAGAGGAAAAUAAUAACAUUAUAUCAGAGUAUUUCUCUGGUAAUUCUUCGAAUCCACUGCAAUCAAAUACCAAACCACUGCAGCAAAAUCAAUCUAGUAUAUUGAGUUUUCAUCUCAGGGAAAAUGAACCAGCAAAGAAUCUUGGCGAACGCAAUGAAUAUCUGAAAAGAAAUGCACGCCAGCUCCCUUCCCUCAAAAGAAGUGCAGGGAAUGUUUCUAGAGAAGAUCUAGGCAAGCACCAUCCUCUUGAUGCAAUCCUUGAGAAUGUACCGGAUGUAUCCCAAGACAGUUUUGGCACCUCUCAAAUGUCAUUGAUGAGCCAAGUUCAAGUUUCUGAACCAAGUGGACAACACAUCGAUUCAUCCUACCAAAUGAAUUCAGUUUGCUGUGAUACCCUUGAUCAGUCAGGAGGAAGGAUGGGCAAUGCAUACUCCUCAACAUCCAAGAAUACACUUAUUCGACAUAGUAGCUCCCCAGCUGGGCUACUCUCUGAACUAGUUGCUGAAGGCCGAGGUACUUUGAAAGUGG | 591 |
| pde-miR946a | UUUCUACGCAGGAUCCUAGGGUUUGGGAUCUAGGGUUUGAAAACGCGCCCAUCUAGCUUAAAUGGUCGAUUUAUGGACGCAUCGUUGGUCACGGGUGUAGGUUCGUGGUCUCUUGUUCUCCACGGUGUGCAUAUUCUCGUGAUUCUUAUCUUUUCGUUCCGAAUAUCAAGCGAUGGCACCAUGACAAGUUUUGCAGUGCUCGGGAGCUGUUUUCUUGCAGUGCGAAGAAAUCGAUGUGUUCAGAGUGUAUAGUUGUGGAUAGAGAAGGGUUAGUAAACGGUAGAAAACCCAUUUAUUGUAUAUCAUUGGCUGUUCGAGAUUAAGCACCUGUAAUUCGAUGGCCAUACUCAUUAAAUGGGUUGCCUGUUUGCUCUGUACCAGCCCUUCUCCUAUCCACAAUUGUACUAGUUUG | 412 |
| pde-miR947 | GGAAGCAGUUAAGCGCAGGCUGCUGCGUGCGUGAUACGCCUAAGGCGCAGCAGCAGAUUCUGAUAGAAGACUCAGGCAAAGCAUUUGUUGUUGGCAGGUUGAAUGCUUUCGGCAGAACCACCAAUAACAAGGGGGGGCGCCUGGUGUCUUGCAUCGGAAUCUGUUACUGUUUCCUAGGCGUCAGGCAAAUCUAAAUCUUUCCUCGCAAAAGCUGAAAUGGCUGUGCAAUAUGGUUUGGAAGGUCUGGUGAAUUUGAUCCGAUAAGGC | 267 |
| pde-miR949a | CAGAGCUUCUCUAGGAAUCAAAUGUGUCUUCCUCUUGAACGCCUUUCACGCACAGGAAGGCCCAUUGGAUUCCCGGGAGAAGCUCUCC | 88 |
| pde-miR949b | GCGCGUGAAGGAGAGCCUCUCCGGGAAUCCAAUGCGCCUUCCUCUUGAACGCCUUUCAACGCGCAGACUGUGCGUGAAAACGCUUUUAGGAGGAAGGUGUAUUGAUUUCCGGGGAAGCCCUGGUUCUCCGUAUUCUUAUUCAUUGGAUGUAUCGAUGGUUUGCAUGUUAGUUUCAAUCAGAGAAAGCAACUGACCGUUUUGUUCCAGAUUC | 211 |
| pde-miR950a | GCCGAUUCAAAAUGAUGGGUAGAUGAGGCCCUACUCCUGUAACCCUAAUUAGGACAAGCAUUUUACACUAUCACUUAUUGCGGACCGUGAUCUGGAGGAGGAAUCGAGCGUGUUAUUGCCCUCGCGGCCACGGUGGUUUAUGAUCGUUUAGGAUGCUCAGAACCACCGUGACCCCGACGAGAAUACUGGGCUCUCCUUCGGUUACAUCGGUUUCGGAAAACUUGACUCCUCAAAUAGAAACAGCUUUUGCUACAGGAUGACUGGUAUUGUUUAUUGCUCUGCCGGUUAAUAUGGAAGAAGAAGCGAGCGUCUUAUUGCCGUCCGGGCCACGAUGGUUUAUGAUCGUGUAAGACGUUCAGAACCAUCUUGACUCUGACGAAAAGAAUACCCUCGCAUUUGGUUACAUUGAUUUGGGAAAACUUGGCUCCUCAAAUAGAAGCAGCUAUUGCAACACCCUUUGCCCAUCUUGGGAAUUCCUUUAGCAAACUGUAUGUUGAAUCAAAAUAUUGUAUUUAAUAGAUCCAAAAAUAACAGGGAUAGAGGUAGGGUCAAGAUGAAGGUUAAAGUUAAGUUGAUUUUAUCUUAAU | 500 |
| pde-miR951 | GAAGCGAUGGUGUUCUUGACGUCUGGACCACGUGGGUUUGCUUUACGUUGGGCAUGAAUAAACAAUCAUCUACCGCGGUUCAGUCAUCAAGAACACCUUUGCUUUCAUUUACAUG | 115 |
| pde-miR952a | GCGAGCUAUCGAAGGAGAGAACCAGUGGCGUAUUGAACAGAGCAUGCCAUUGGUGGAGUAAGUACGUCAAGGCACGAAACAGAAUUAAUUUUGAUUAACGUUUUAUUAACCUUCAACUCUGCAUUGGCCUAUGGCAGUUCCUCAAGGUCACAUCGAUGGACAACUCUGCAUUGGCCUAUGGCAGUUCCUCAAGGUCACAUCGAUGGACUGGGGCGUGAUACAAGGCGUAGUCCGCUGGUUUCUGCCACCGUUAGGACUCUCCCCAACCUAUUCUCAGCUGCAACAUUUACAACUGCAACAUUCACAAGUUAUUUACAAUGUUGCAGCCGAGAAUAAAAUGGGAAGACUCCAAAGCGUCGCAACAACCAGCUUUGUAUCAGCCCCCCAACUGGUGUGAGUUUGGGGAACUGCCACAUGCCAAGCCAGACUUGCAUGCUAAUAAAACAUUAUUCGAAAUUGCUUCUAUUUUGUUUGUUGACAUACUUCCUCCGCCAAUGACACGCUCAGUUCAAUUUGCUGAUGGUUC | 526 |
| pde-miR952b | GCGAGCUAUCGAAGGAGAGAACCAGUGGCGUAUUGAACAGAGCAUGCCAUUGGUGGAGUAAGUACGUCCAGGGACGAAACAGAAUUAAUUUUUUUGAUUAACAUUUUAUUAACCUCCAACUCUGCCUUGGCCUAUUGCAGUUCCUGAAGGUCACAUCGACUGGGGCGUGAUACAAGGCGUAGUCGGCUGGCUGCUGCCACCGUUGGGACUCUCCCAACCUAUUCUCAUCUACAACGUUAAAAAGUUACUUACAUUGUUGCAGCCGAGAAUAAAAUGGGAAGACUCCAAAGGGUCGCACCAACCAGCCAAUUGCGCUUUGUAUCAGCGCGCCAACUGGUGUGAGCUUUGGGAAUUGCCAUACGCCAAGCCAGACUUGCAUGCUAGUAAAACAUUAUUCGAAAUUACUUCUAUUUUGUUUGUUGACAUACUUCCUCCGCCAAUGACACGCUCAGUUCAAUUUGCUGAUGGUUC | 472 |
| Pde-miR952c | GCGAGCUAUCGAAGGAGAGAACCAGUGGCGUAUUGAACAGAACAUGCCAUUGGUGGAGUAAGUACGUCAAGGCACGAAACAGAAUUAAUUUUGAUUAACGUUUUAUUAACCUUCAACUCUGCAUUGGCCUAUGGCAGUUCCUCAAGGUCACAUCGAUGGACUGGGGGCGUGAUACAAGGCGUAGUCCGCUGGUUUCUGCCACCGUUGGGACUCUCCCCAACCUAUUCUCAGCUGCAACAUUCACAAGUUAUUUACAAUGUUGCAUCCGAGAAUAAAAUGGGAAGACUGCAAAGCGUCGCAACAACCAGCUUUGUAUCAGCGCCCCAACUGGUGUGAGUUUGGGGAACUGCCACAUGCCAAGCCAGACUUGCAUGCUAGUAAAACAUUAUUCGAAAUUACUUCUAUUUUGUUUGUUGACAUACUUCCUCCGCCAAUGACACGCUCAGUUCAAUUUGCUGAUGGUUC | 465 |
| pde-miR1310 | AUUAGAGGCAUCGGGGGCGUAACGCCCUCGACCUAUUCUCAAACUUUAAAUAGGUAAGAGGGUGCGGCUGCUCCAUUGAGCCG | 83 |
| pde-miR1311 | UGGCCGCCUCUGCUUAUGGGCUUGGUAGGAACAGGCGGACUGGCAUAACUCCGCCCAUAUUUUACUGUCAGAGAUGGUCAGAGUUUUGCCAGUUCCGCCCAUUCCUACUGUGCCUUUUAAGUAAUUUUAGAAAUGGAGCAAUUCGAUAUGCUCGGUGCCUUUA | 163 |
| pde-miR1312a | CAUCCAUAUCGCUAUAUCUCUCCUAAUUAGGUUGAAUCGAAACAGCCAACACUUUCAGUUGAUUCAAAUAUUAGUUUGUUUCUGCAUAAUUUGGAGAGAAAAUGGCGACAUAGAUGUUUUCGGCGAUAGUUCUGAAGAAAACGAAAUGUAACGGAUAGCGUUCCAGGGAAGCUCCACCGAGAUCAAAUACAGAUUAACUCUGCAAGAGGAAGCUGGAUGCUGUACUCCAAUACGCUGCAGG | 241 |
| pde-miR1313 | UCGUGAUGGUAUUCUACCACUGAAAUUAUUGUUCGAAAUAACACCUGCAAUUCAUUGAUGUAUACCACCGUGGUAUGGCAUCCUUCAGCGUUUCGCCAAACUAACCUAGUUCCAGGGUUGCAACACAUGGCAUGGUGGAGGAUCUAUUGGGAAAUAACCAAGUUGCAUGAGUGUAUUUAUCCAACAAUAAUUUCAGUGGAAGACUUCCAUCGAUCUGGAAGACUCAU | 227 |
| pde-miR1314a | GUCAUGUUGGUUAGAUGAAGGAAUUCUUCAACAUUAGAGGCCGAUGUGGAGUUAUGCUCUUGACCGGCCUCGAAUGUUAGGAGAAUGUUUUCCUCUACCUAAGCA | 105 |
| pde-miR1448 | GUCAUGGGGUCUUUAGGCUUUGGAGGAUUUGGAAAGGCUUAGUCAUUCUUUUUACCGAGGAUAUUCGAUUUUCUAAGAACCUUUCCAACGCCUCCCAUGCCUAUAGUCCUCAUUGUACAUGUCACCCGAUCGCAG | 135 |
| pde-miR2118a | GUCAUGGGGUCUUUAGGCUUUGGAGGAUUUGGAAAGGCUUAGUCAUUCUUUUUACCGAGGAUAUUCGAUUUUCUAAGAACCUUUCCAACGCCUCCCAUGCCUAUAGUCCUCAUUGUACAUGUCACCCGAUCGCAG | 135 |
| pde-miR2118b | UUUGUUGAUUUUGAGAAGGGAUCUGUCAUGUGGAUGGAAGUCUUGAGGAGUGGGAGGGUAGGAGAAGGCUCUGUGGUGAGGUUUCAGUCAUAAUCUCAUCAGUCUUCCCUAUUCCUCCCAUUCCUAUUGCCUCCCUUCACAAUGUAACACAGUACACAGCCUGUUAAAGAAACGAAUAUGGCUAAAUAUCAUUCUGUUUUGUGGGUAUUAUGGGGAUGUCCUGUAGUUUUAAGGCCAUCAGGUCUCUGUGGGUUGGAAAUACCUACGCAGUAAUUUAUGAUCUGAUGACAUCUAUGCACUGUAACAGUCUUCCCAACUCCACCGAAACCCAUGACCUUCUUCACUACACAUCCCUUUUGUGCCCGCUCUAUUAACUCUGGCGCUGUUACUCUCCAGAUCUGCAAACAUGGCACAGGGGCAUGGCUGCAACAUACAGUUGAUUGUAAGCCUGUCCCUGCUUUAUGUCGUGAAUUUAUUUAUUUUUGCAGGGGACAUGGAGCUUAUUAUACUGUGACCAAGAUCUGGAUUUGGGUGUAUGGCUUGGAUGUUAGAACAAGAAUUAGUUCUGGAUUUGGGUGUAUGGCUUGGAUGUUAGAACCAGCAUUAGUUUUGUGUUUCCUUUCUUAGAGUACCAUGUAAUAAUUGUAGAGAAGUCAAUUUGAUUGUAUAAAUUGUUUGAUAGUAGGGAUGCCUGUUUAGAGAGGAUUAUGGUAUGUAAGAACCUUUAUUUUAAGGUCUCAACCUAUCAUAUUAAGCUGUUGAAAUUAGUAAUAACAGAUCUCAAGUUCAGGCAAAUAAAAAUGUCUCCG | 809 |
| pde-miR3701 | GUGAAAAGUUCUCGUGGAAAGCUCUCUUCAUUUCUAAAAGGAGAACACAUUUAUUCCCAAAUCCUAUCAAAUAACAUAUGGGUUUGCCCGCCACAGAUGAAGGGUUGGAGUUGUUCGGUAGAAGAGUUUCAUCUGUUGUAGGAUAUGGAGGAUUUUCCAAAAUAUUUGCCAUCAAAUUAAAAGACUAUAGUGGUUUCAUUGCAAUUUGUUUGGCACUAUCCUCUAUAUCCUUCUGCCCUAAAAUUCUUCUCCUGAACAAUGCCCACCCUUCAUCUCUGGGCAUAUUUCCAUGUCUCUCUCCCAGAUCGCUUCUAUUACGAGAUAAGUUCACUCAUUUCUUCCCUGUGUAACAACUUUAUCUUUUGUGCUAGUUUUUUGUUUUUAC | 385 |
| pde-miR3704a | CGGAGACAUUUUUAUUUGCCUGAACUUGAGAUCUGUUAUUAGUAAUUUCAACAGCUUAAUAUGAUAGGUUGAGACCUUAAAAUAAAGGUUCUUACAUACCAUAAUCCUCUCUAAACAGGCAUCCCUACUAUCAAACAAUUUAUACAAUCAAUUGACUUCUCUACAAUGAUUACAUGGUACUCUAAGAAAGGAAACACAAAACUAAUGCUGGUUCUAACAUCCAAGCCAUACACCCAAAUCUAGAUCUUGGUCACAGUAUAAUAAGCUCCAUGUCCCCUGCAAAAAUAAAUAAAUUCACGACAUAAAGCAGGGACAGGCUUACAAUCAACUGUAUGUUGCAGCCAUGCCCCUGUGCCGUGUUUGCAGAUCUGGAGAGUAACAGCGCCAGAGUUAAUAGAGCGGGCACAAAAGGGAUGUGUAGUGAAGAAGGUCAUGGGUCUCGAUGGAGUUGGGAAGACUGUUACAGUGCAUAGAUGUCAUCAGAUCAUAAAUUACUGCGUAGGUAUUUCCAACCCACAGAGACCUGAUGGCCUUAAAACUACAGGACAUCCCCAUAAUACCCACAAACCAGAAUGAUAUUUAGCCAUAUUCGUUUCUUUAACAGGUUGUGUACUGUGUUACAUUGUGAAGGGAGGCAAUAGGAAUGGGAGGAAUAGGGAAGACUGAUGAGAUUAUGACUGAGACCUCACCACAGAGCCUUCUCCUACGCUCCCACUCCUCAAGACUUCCAUCCACAUGACAGAUCCCUUCUCAAAAUCAACAAA | 764 |
| pde-miR3704b | CGGAGACAUUUUUAUUUGCCUGAACUUGAGAUCUGUUAUUACUAAUUUCAACAGCUUAAUAUGAUAGGUUGAGACCUUAAAAUAAAGGUUCUUACAUACCAUAAUCCUCUCUAAACAGGCAUCCCUACUAUCAAACAAUUUAUACAAUCAAAUUGACUUCUCUACAAUUAUUACAUGGUACUCUAAGAAAGGAAGCACAAAACUAAUGCUGGUUCUAACAUCCAAGCCAUACACCCAAAUCCAGAACUAAUUCUUGUUCUAACAUCCAAGCCAUACACCCAAAUCCAGAUCUUGGUCACAGUAUAAUAAGCUCCAUGUCCCCUGUGCCAUGUUUGCAGAUCUGGAGAGUAACAGCGCCAGAGUUAAUAGAGCGGGCACAAAAGGGAUGUGUAGUGAAGAAGGUCAUGGGUCUCGGUGGAGUUGGGAAGACUGUUACAGUGCAUAGAUGUCAUCAGAUCAUAAAUUACUGCGUAGGUAUUUCCAACCCACAGAGACCUGAUGGCCUUAAAACUACAGGACAUCCCCAUAAUACCCACAAAACAGAAUGAUAUUUAGCCAUAUUCGUUUCUUUAACAGGCUGUGUACUGUGUUACAUUGUGAAGGGAGGCAAUAGGAAUGGGAGGAAUAGGGAAGACUGAUGAGAUUAUGACUGAAACCUCACCACAGAGCCUUCUCCUACCCUCCCACUCCUCAAGACUUCCAUCCACAUGACAGAUCCCUUCUCAAAAUCAACAAA | 736 |
| pde-miR3712 | GAGAUGUGAUCAAGAUCAGACUCCCAAAUCCAACAGCAUUUGAGGUCUUUGCUCGAUUAAUAUCAUCCA | 69 |
