# Supplementary material for: Transcriptome-wide identification and characterization of miRNAs from Pinus densata
Source: BMC Genomics. 2012 Apr 6;13:132. doi: 10.1186/1471-2164-13-132 (PMC3347991; doi:10.1186/1471-2164-13-132)
Supplement: Additional file 3 — Predicted hairpin structures of P. densata miRNA precursors. The hairpin structures of P. densata miRNA precursors were predicted by MFOLD. Mature miRNAs were marked in yellow. [file 1471-2164-13-132-S3.PDF]

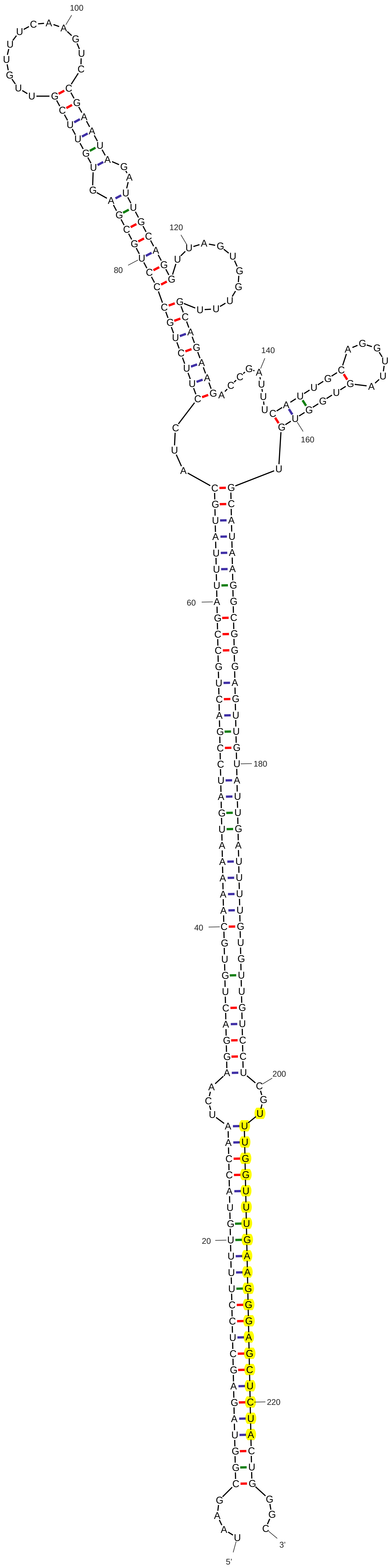

dG = -94.74 [Initially -98.00] pde-MIR159a

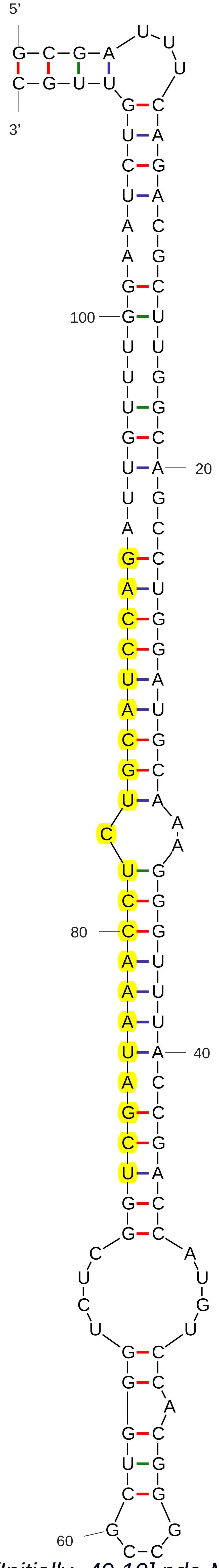

dG = -49.10 [Initially -49.10] pde-MIR162a

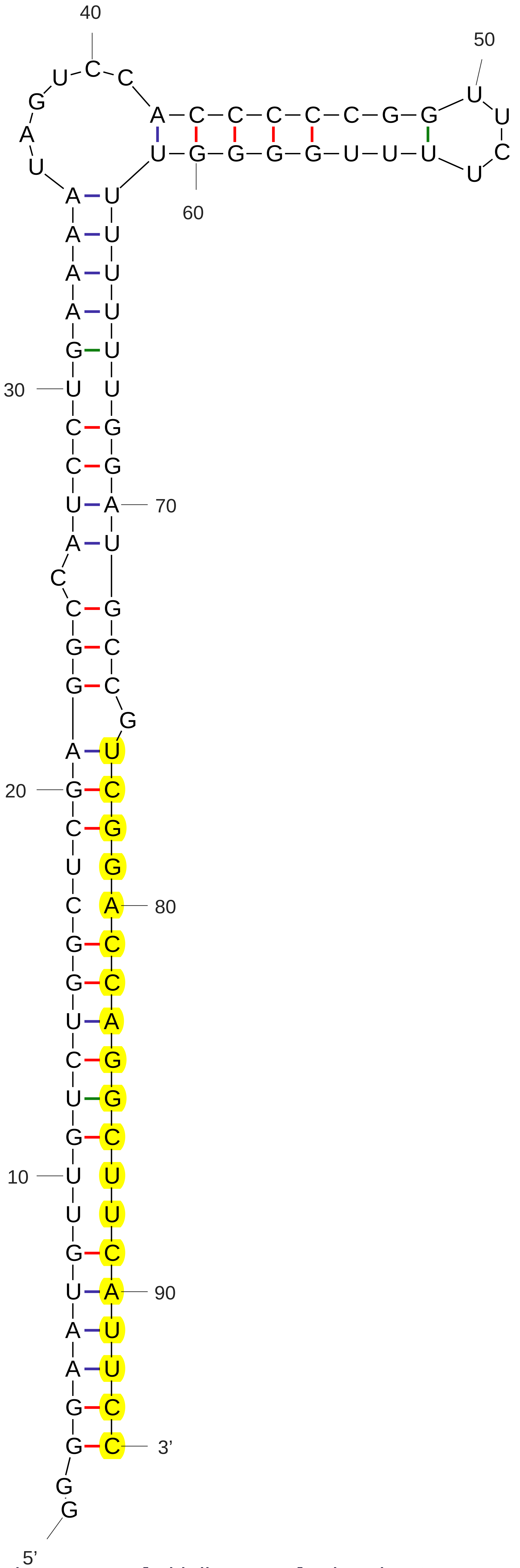

dG = -41.90 [Initially -41.90] pde-miR166a

Output of sir_graph (©)
mfold_util 4.6

Created Tue Feb 28 21:22:07 2012

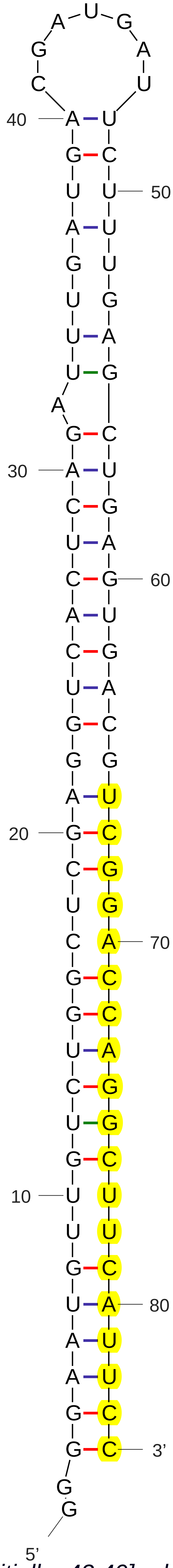

dG = -43.40 [Initially -43.40] pde-miR166b

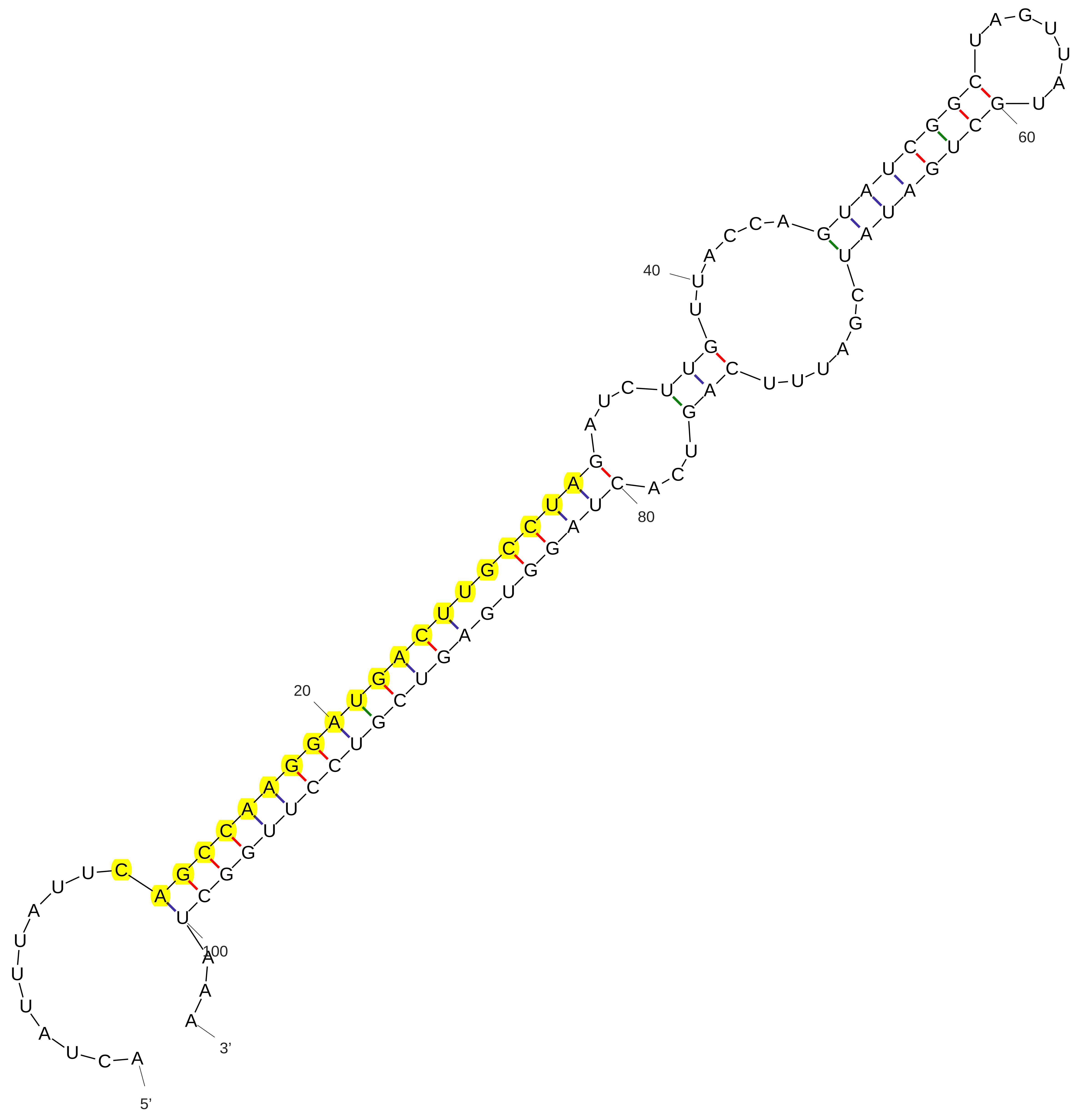

dG = -48.80 [Initially -48.80] pde-MIR169a

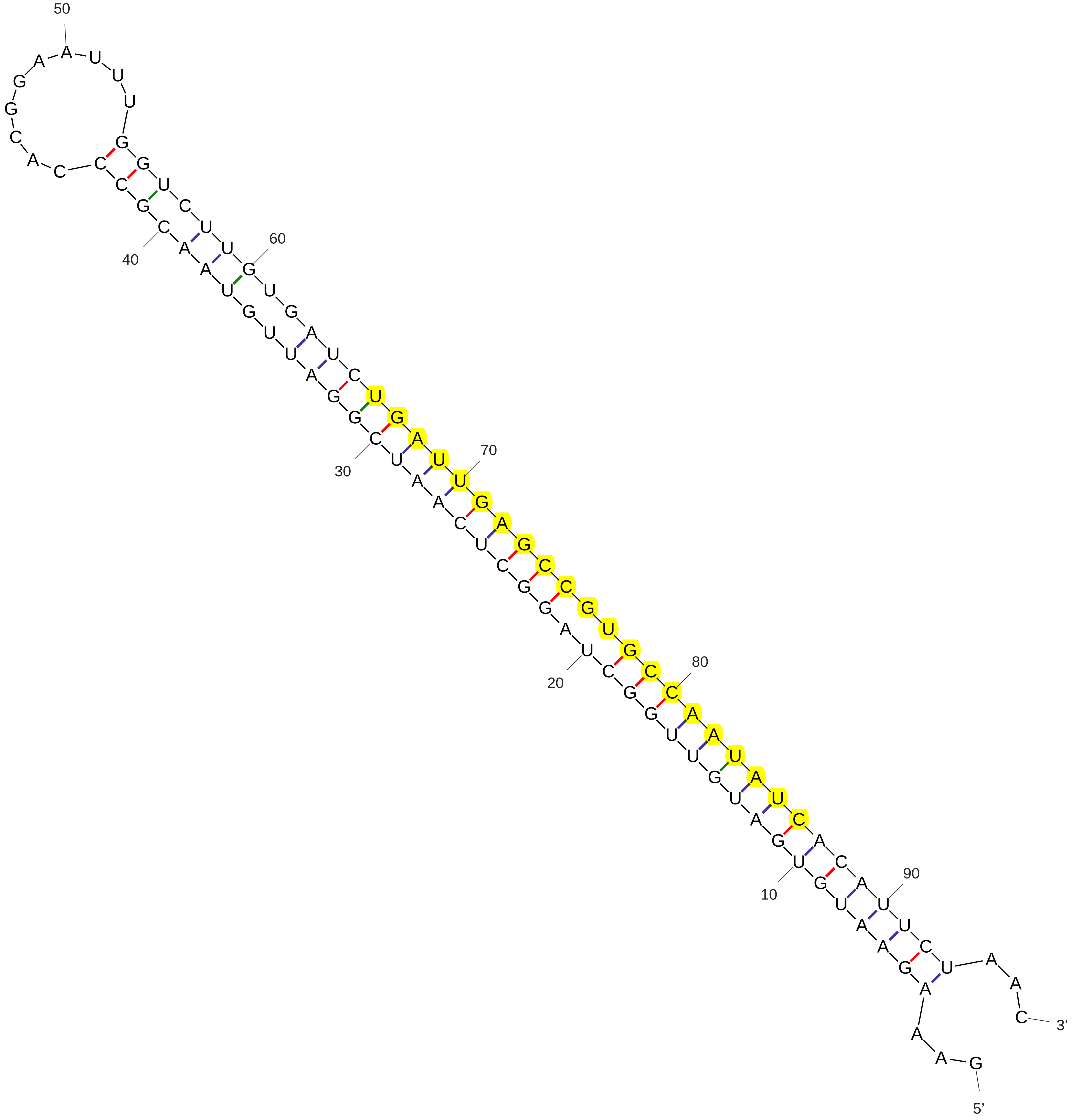

dG = -55.20 [Initially -55.20] pde-miR171a

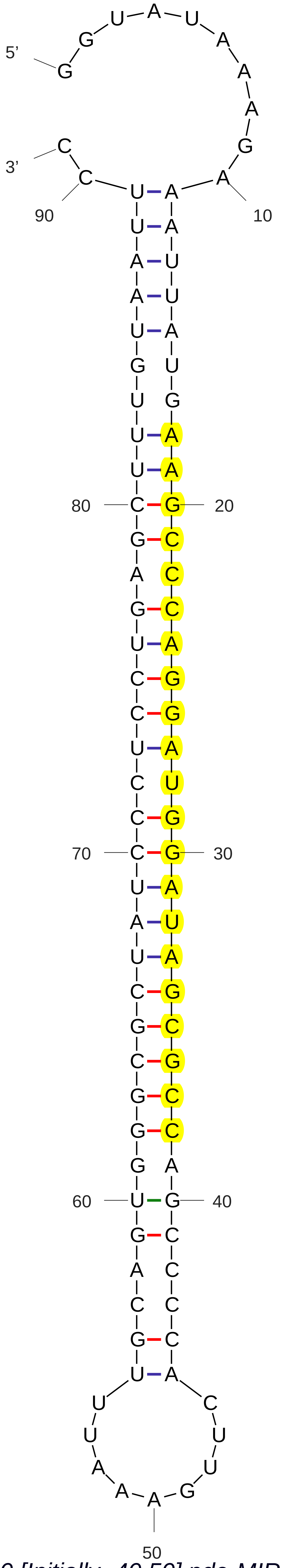

dG = -40.50 [Initially -40.50] pde-MIR390a

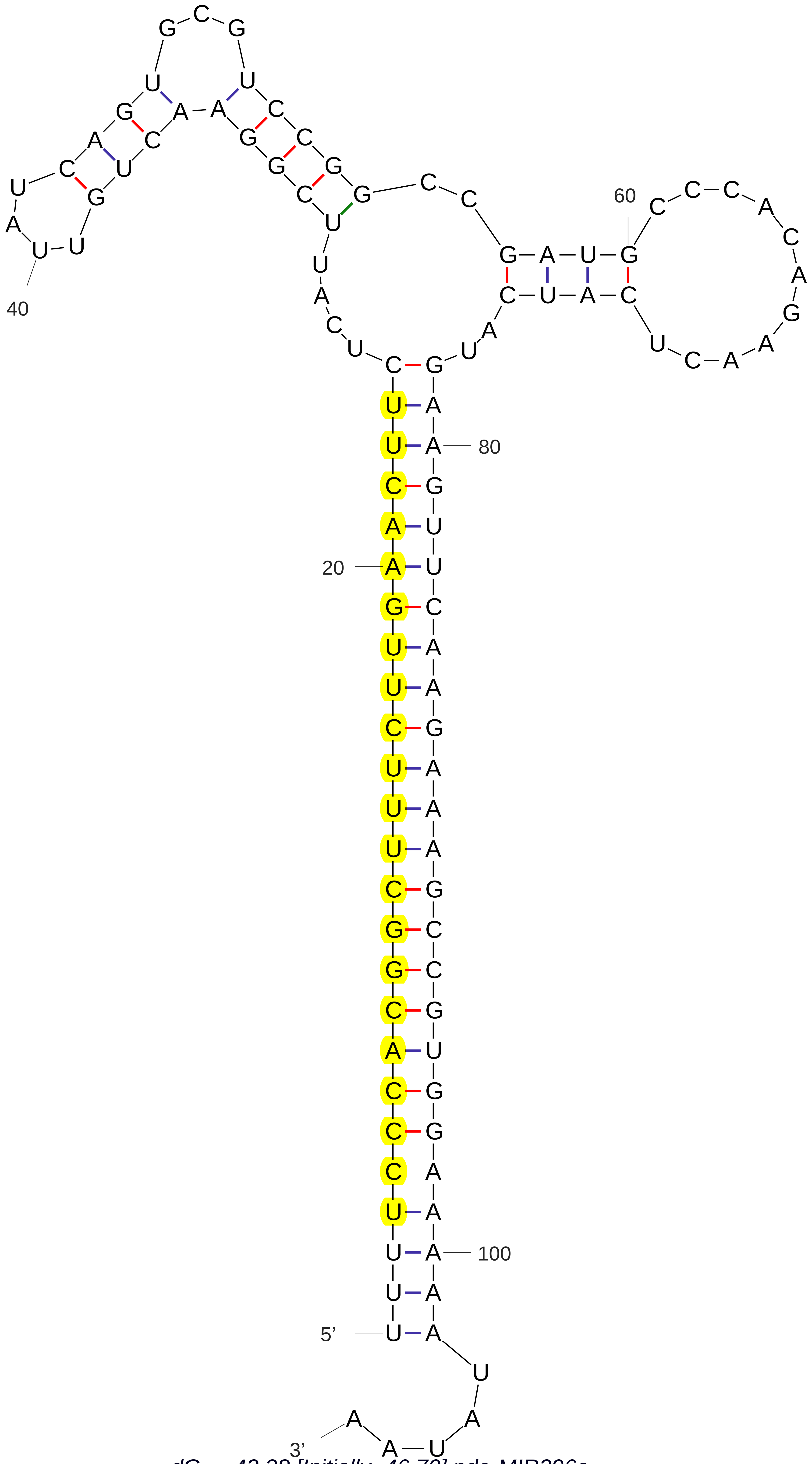

dG = -43.28 [Initially -46.70] pde-MIR396a

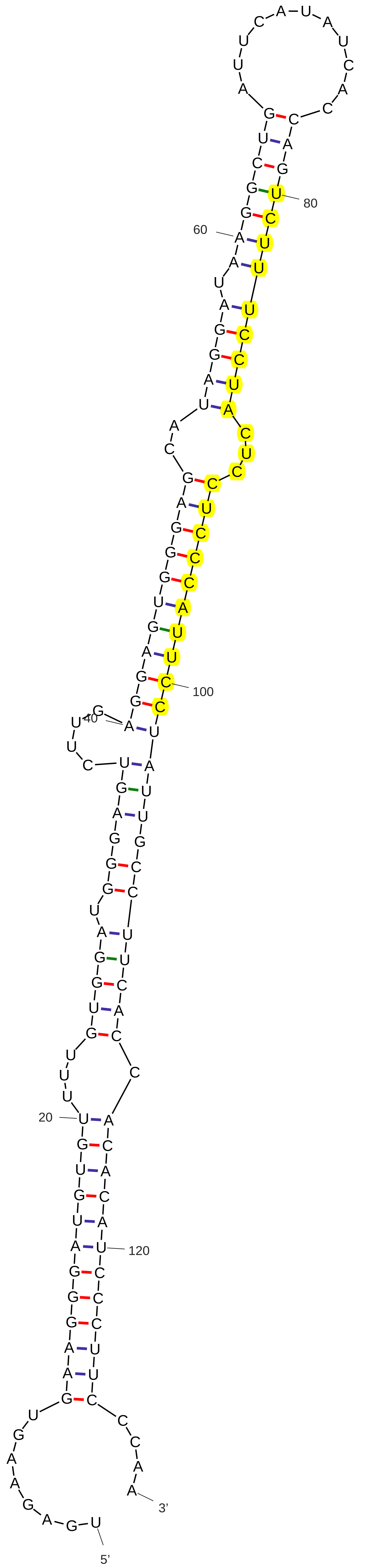

dG = -60.90 [Initially -60.90] pde-miR482a

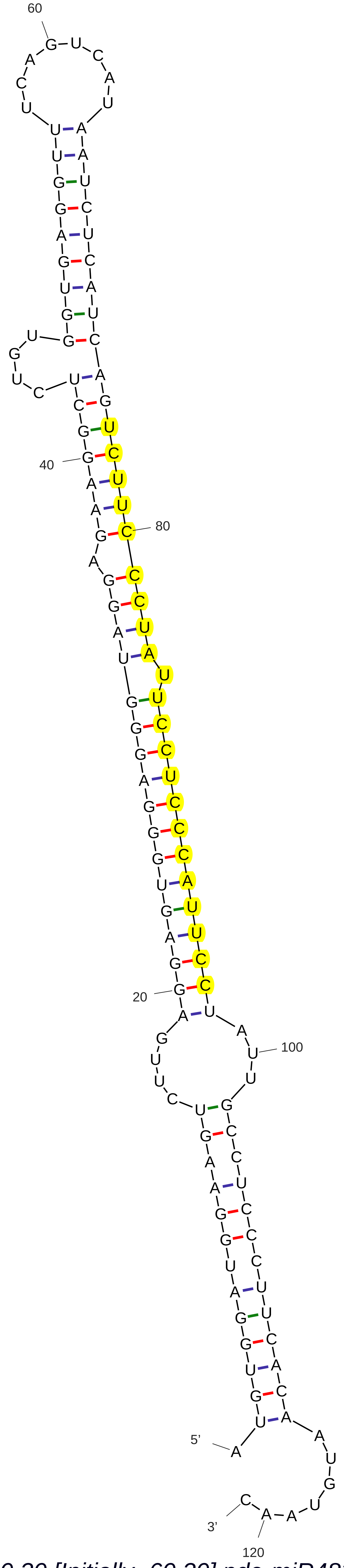

dG = -60.30 [Initially -60.30] pde-miR482b

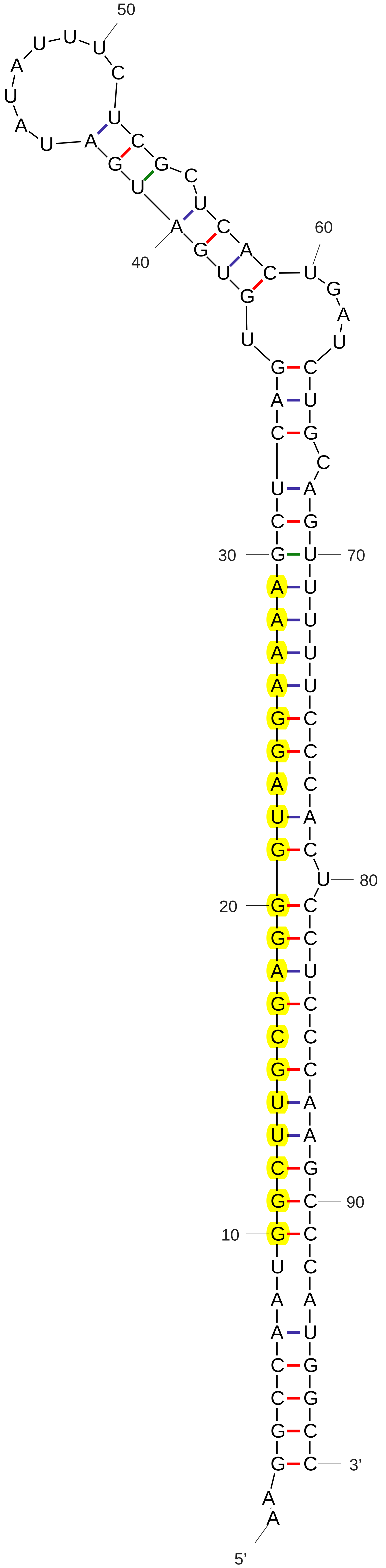

dG = -45.20 [Initially -45.20] pde-MIR482c

Output of sir_graph (©)
mfold_util 4.6

Created Sat Aug 20 03:55:08 2011

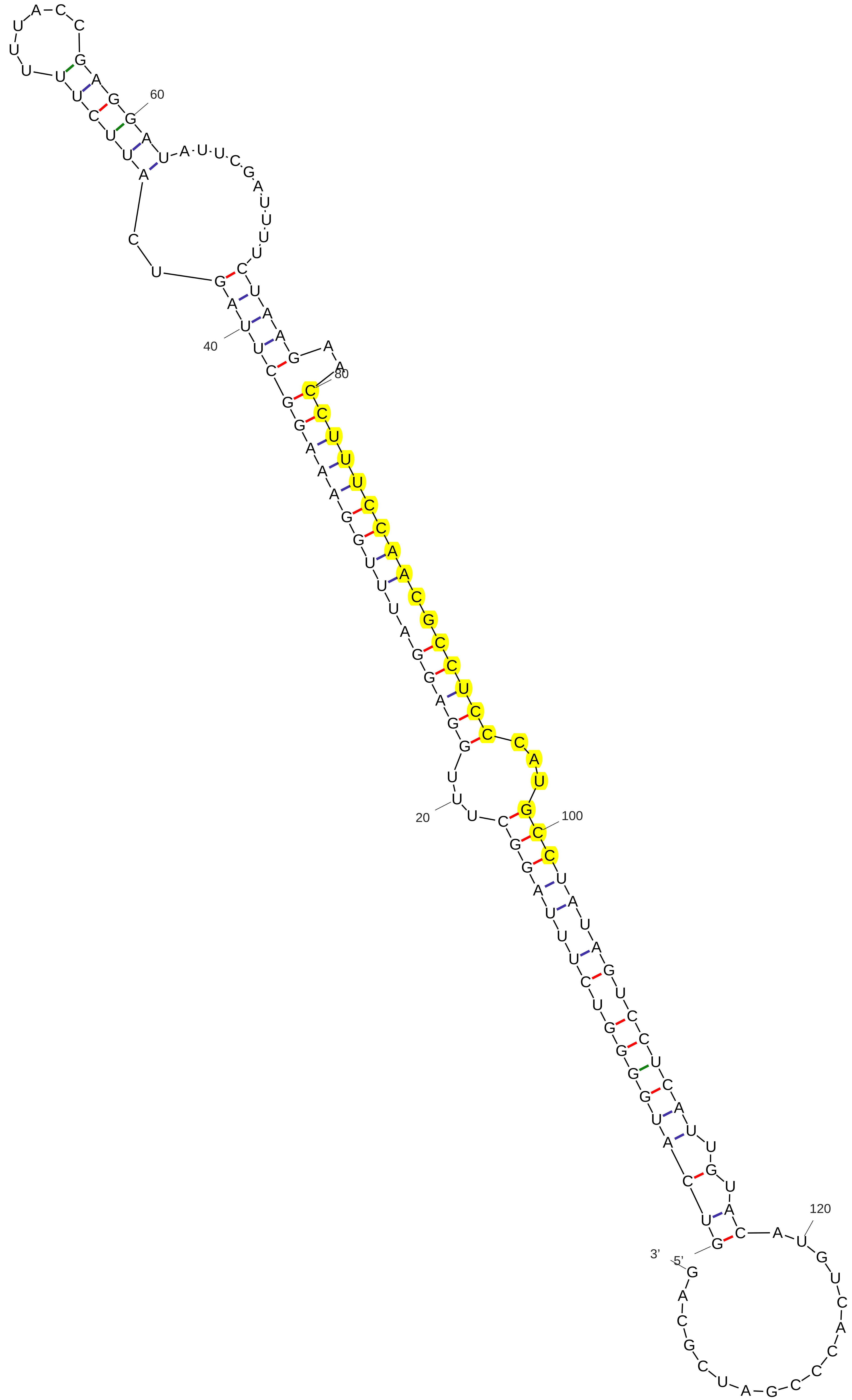

dG = -46.50 [Initially -46.50] pde-MIR482d

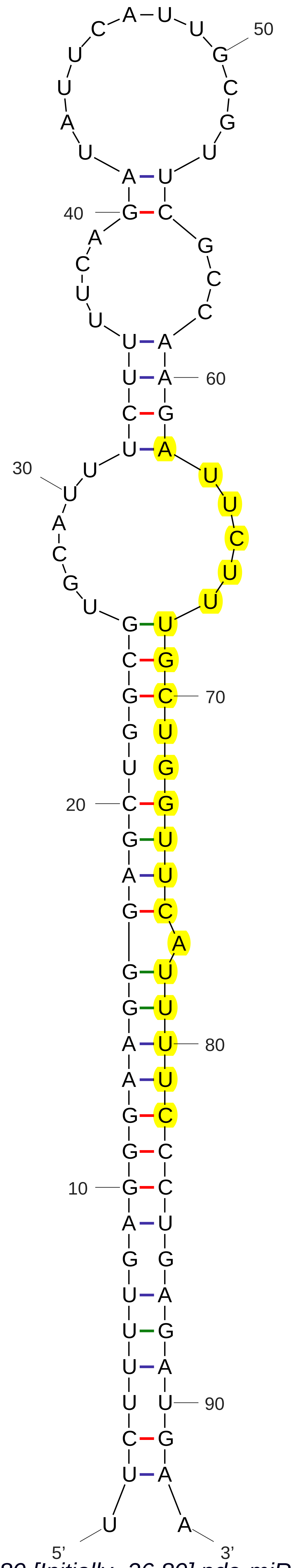

dG = -26.80 [Initially -26.80] pde-miR783

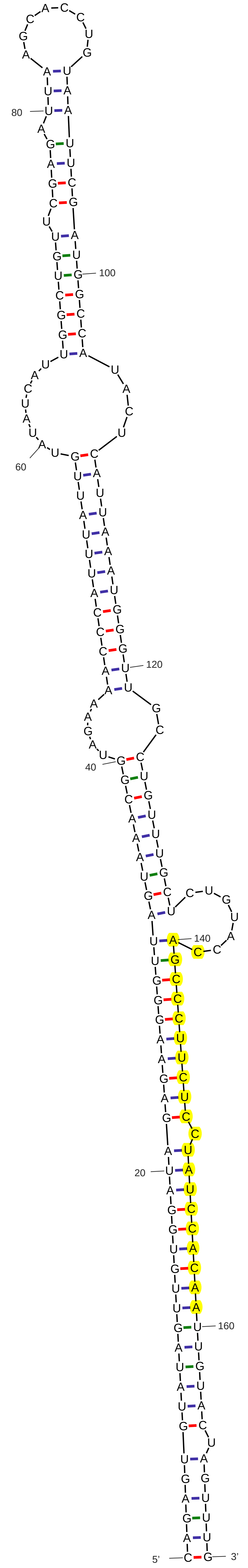

dG = -71.50 [Initially -71.50] pde-miR946a

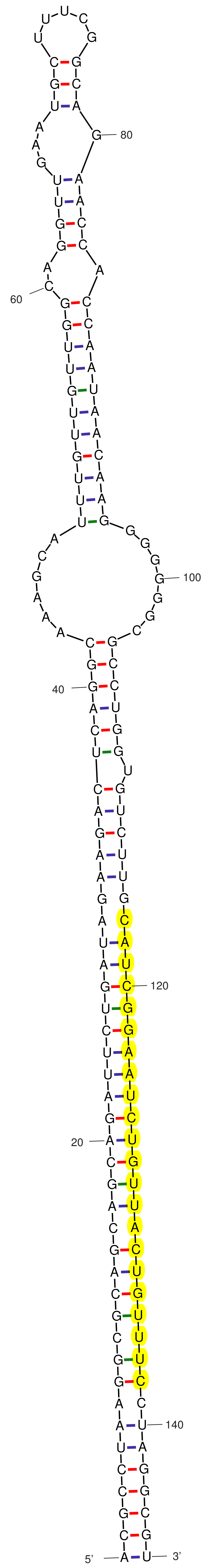

dG = -70.70 [Initially -70.20] pde-miR947

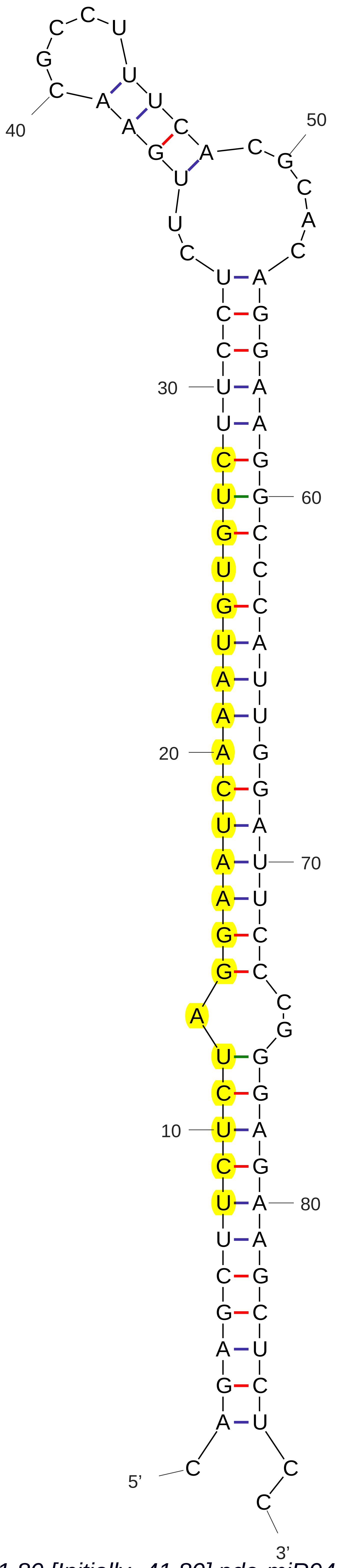

dG = -41.80 [Initially -41.80] pde-miR949a

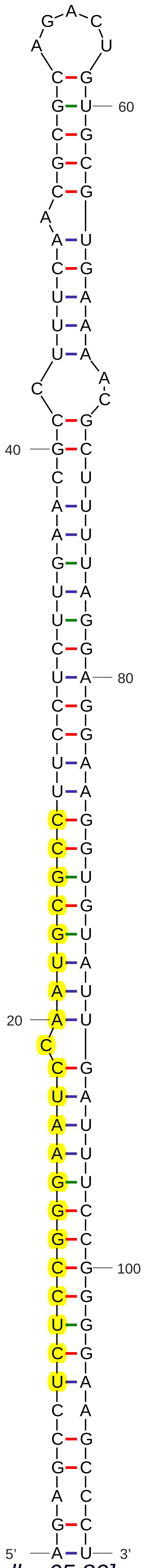

dG = -66.30 [Initially -65.80] pde-miR949b

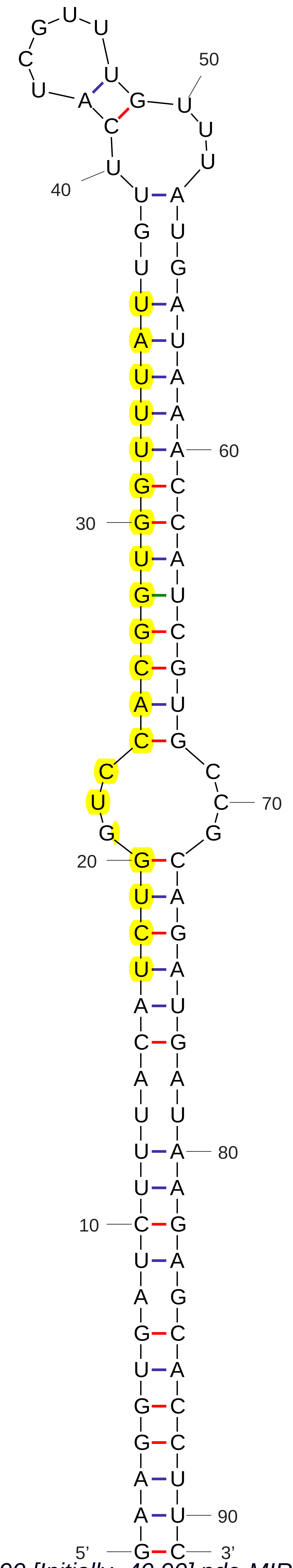

dG = -40.90 [Initially -40.90] pde-MIR950a

Output of sir_graph (©)
mfold_util 4.4
Created Thu Nov 4 21:17:30 2010

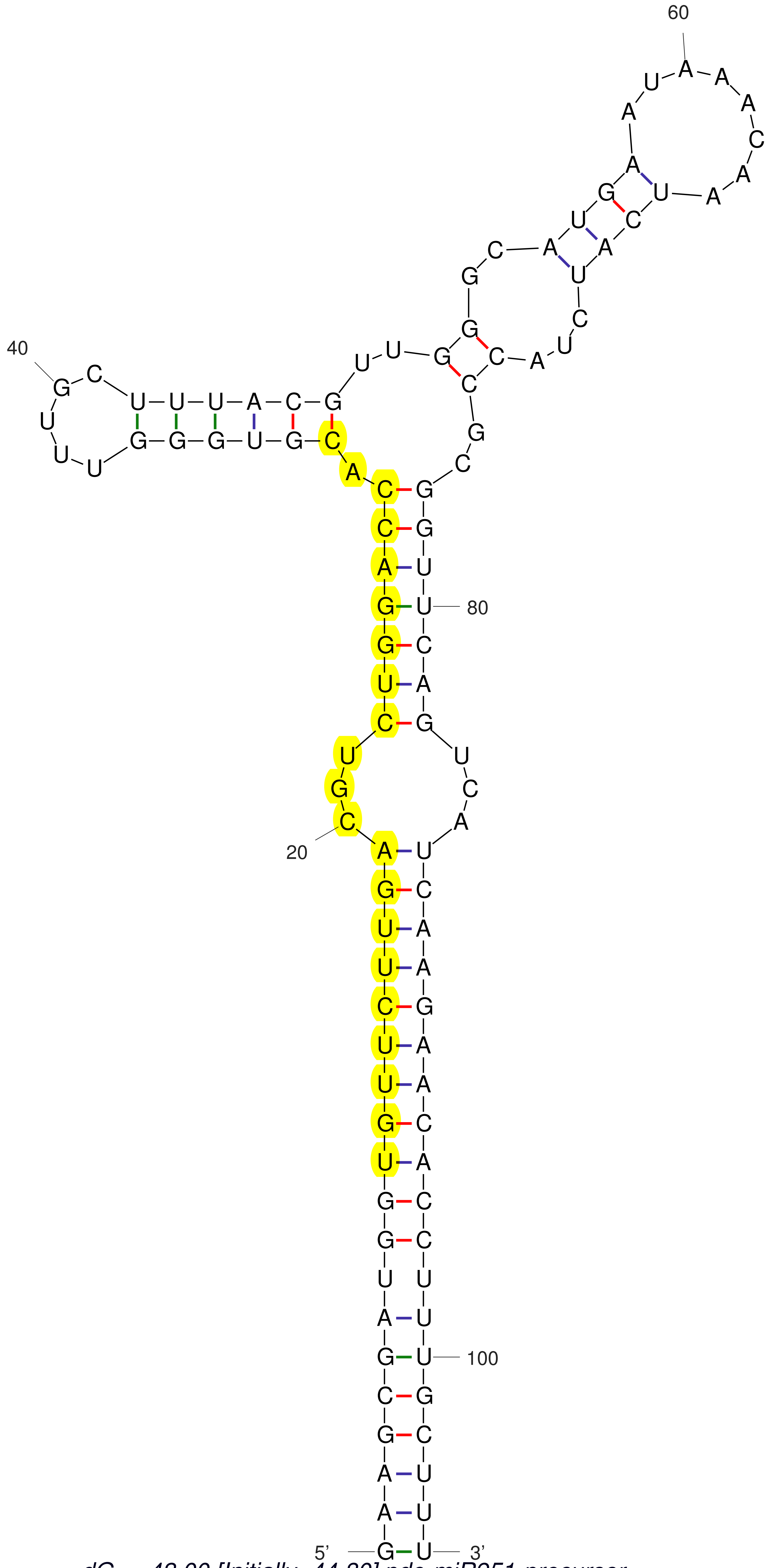

dG = -43.00 [Initially -44.80] pde-miR951-precursor

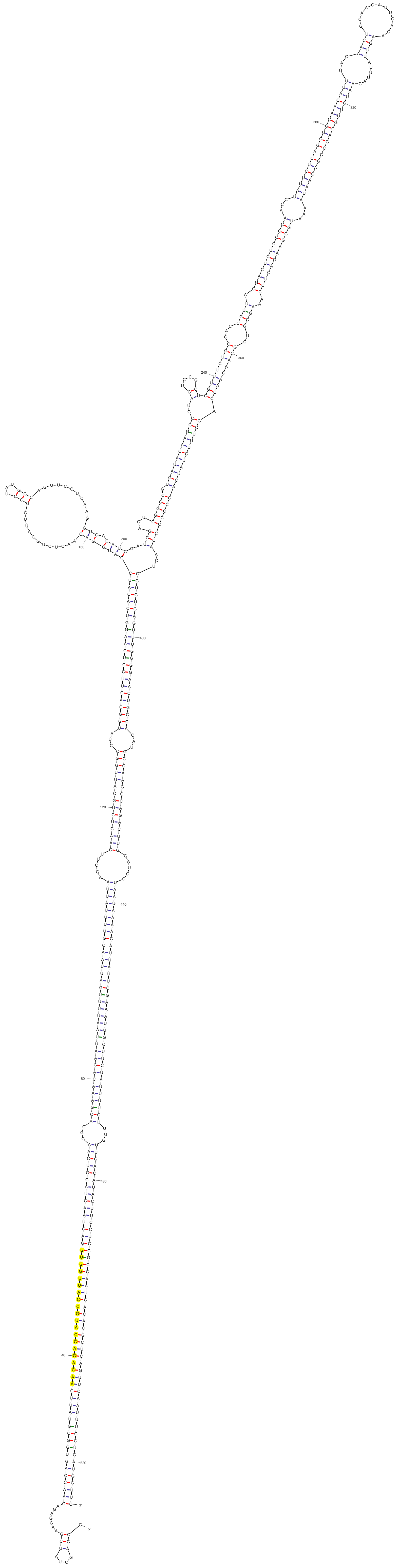

dG = -218.40 [Initially -224.60] pde-miR952a

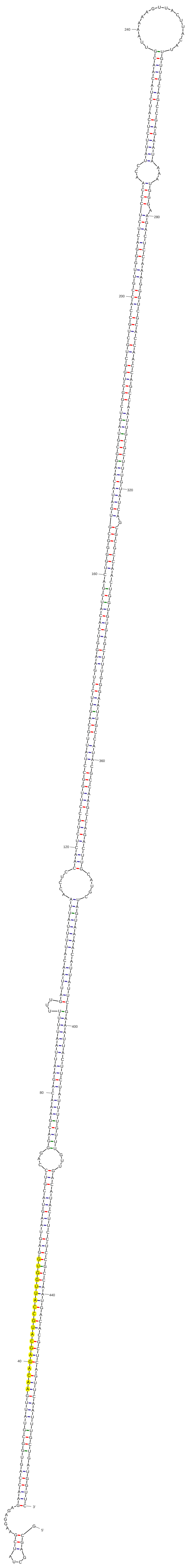

dG = -214.40 [Initially -214.40] pde-miR952b

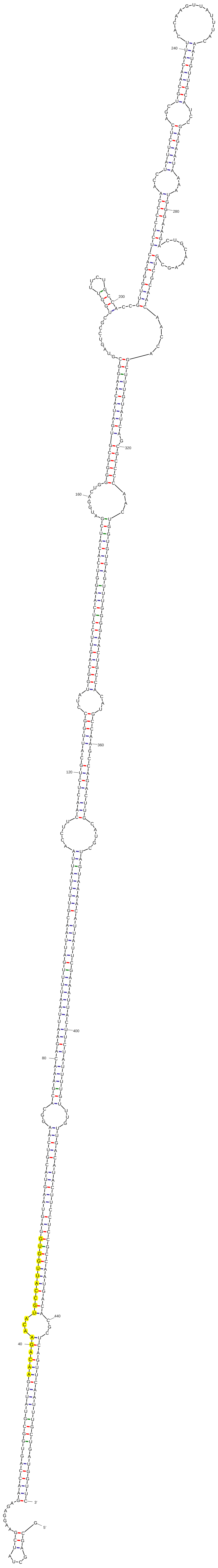

dG = -192.12 [Initially -196.40] pde-miR952c

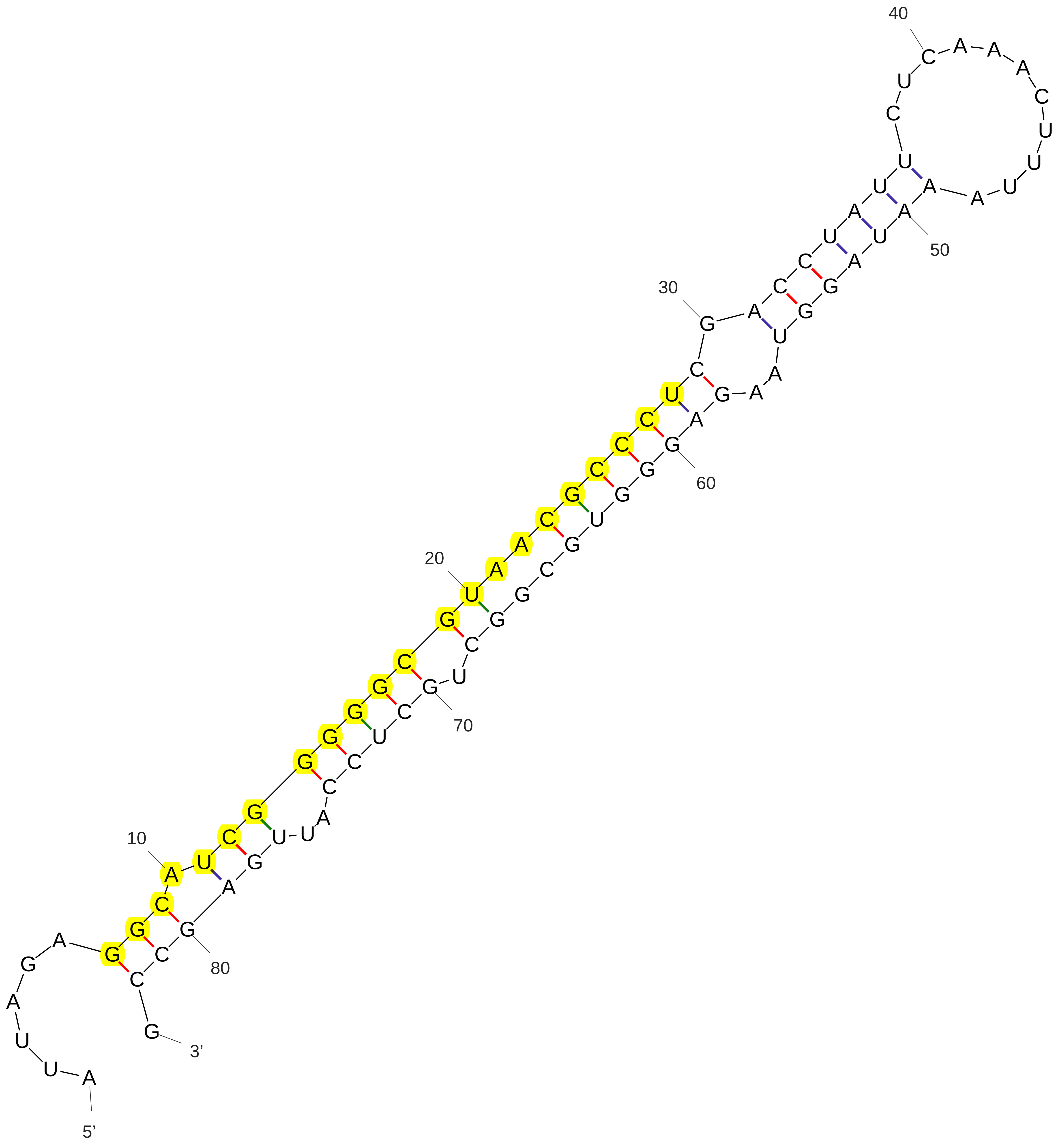

dG = -35.00 [Initially -35.00] pde-miR1310

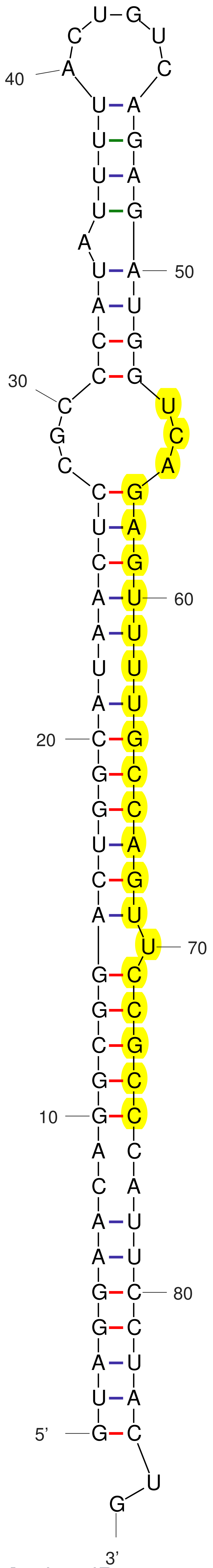

dG = -43.40 [Initially -43.40] pde-miR1311-4157500-anti-precursor

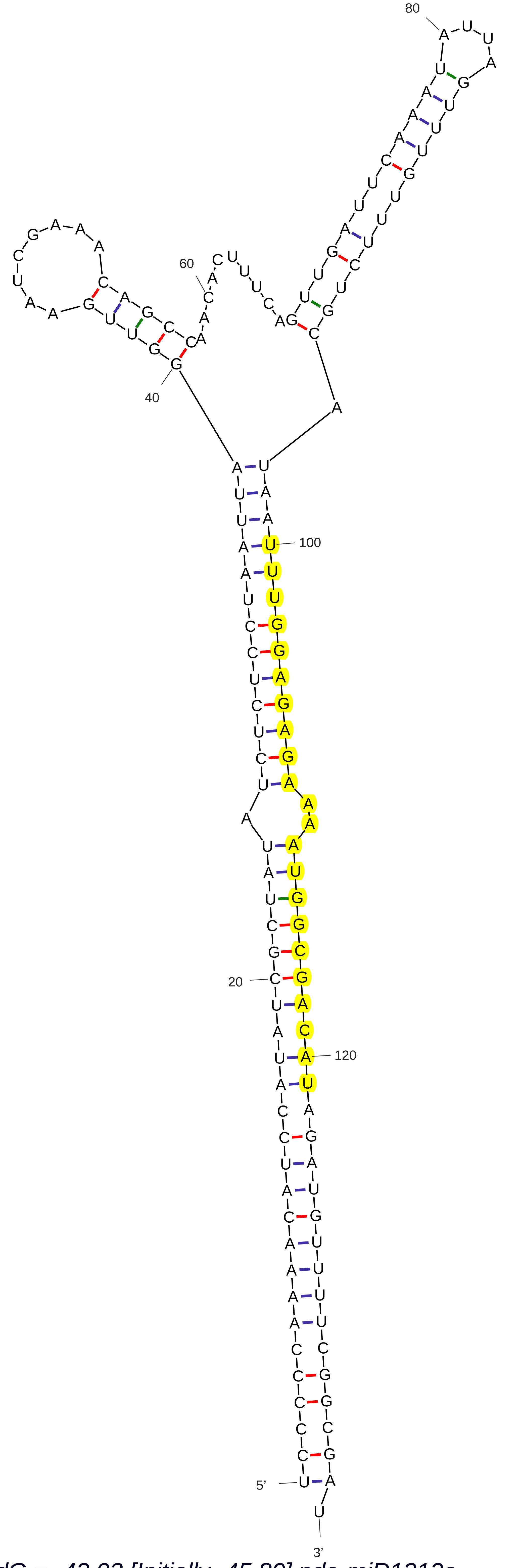

dG = -43.03 [Initially -45.80] pde-miR1312a

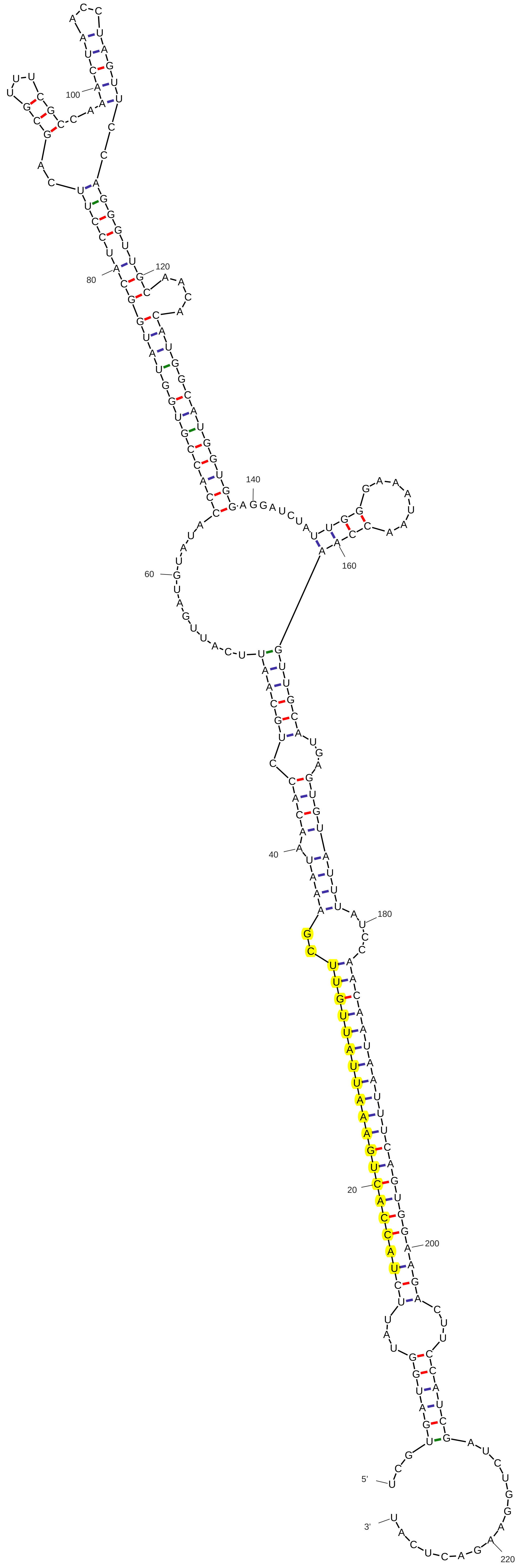

dG = -66.72 [Initially -72.50] pde-miR1313

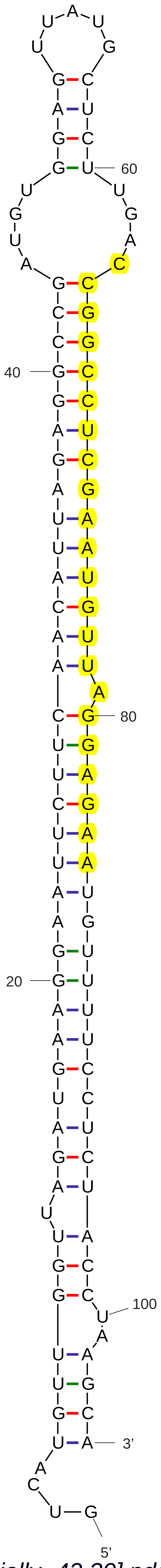

dG = -42.30 [Initially -42.30] pde-MIR1314

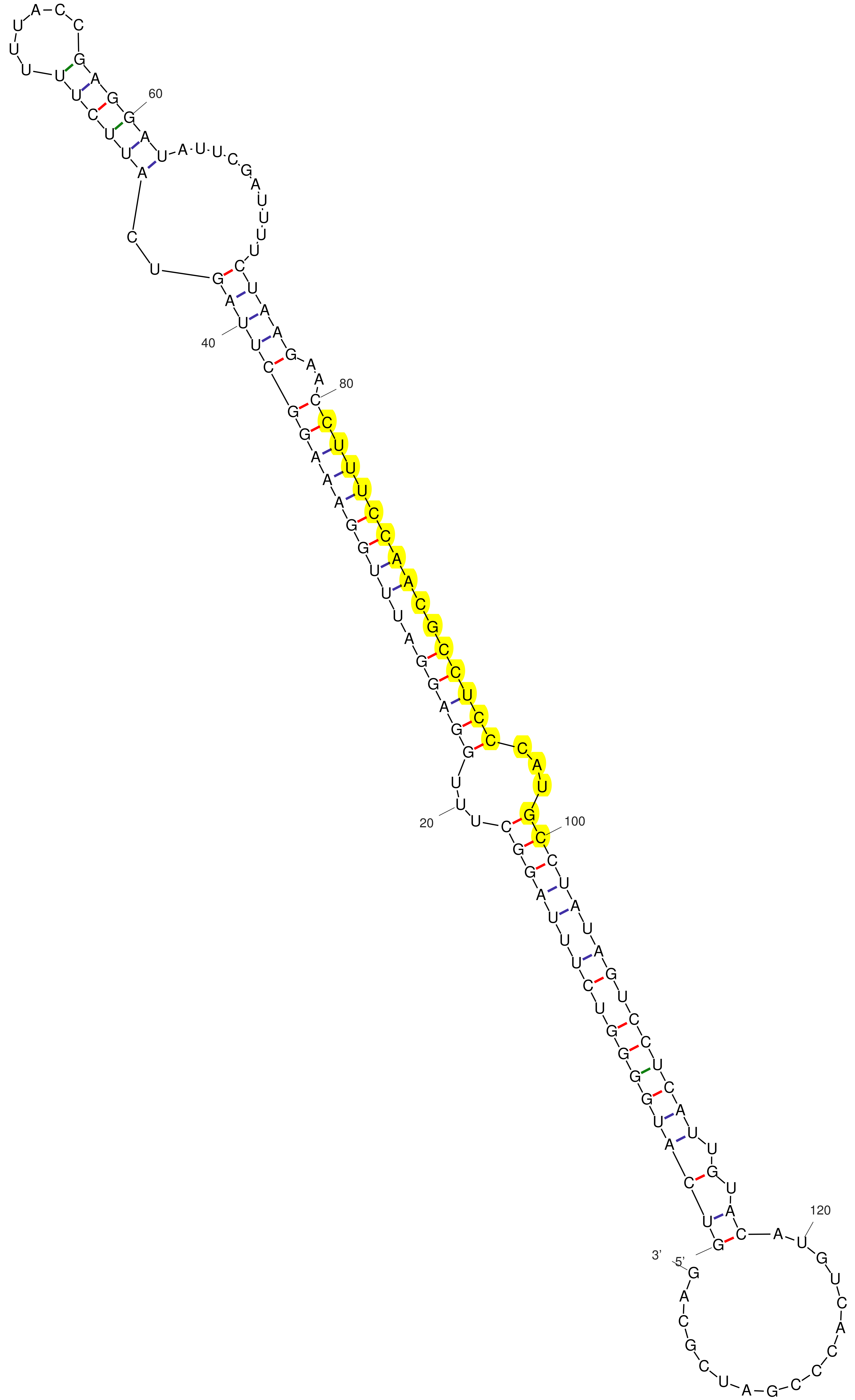

dG = -46.50 [Initially -46.50] pde-miR1448-3959581

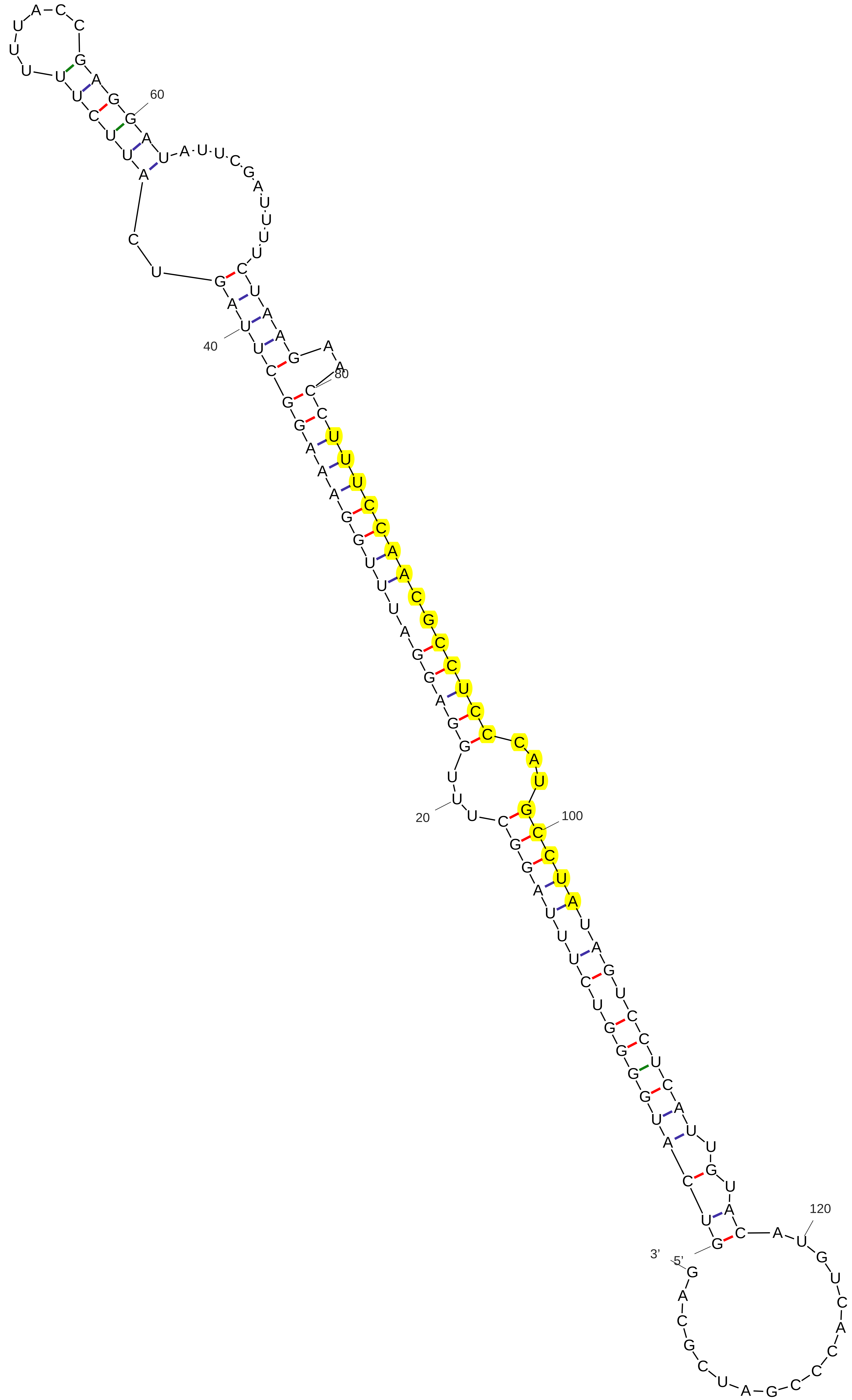

dG = -46.50 [Initially -46.50] pde-MIR2118a

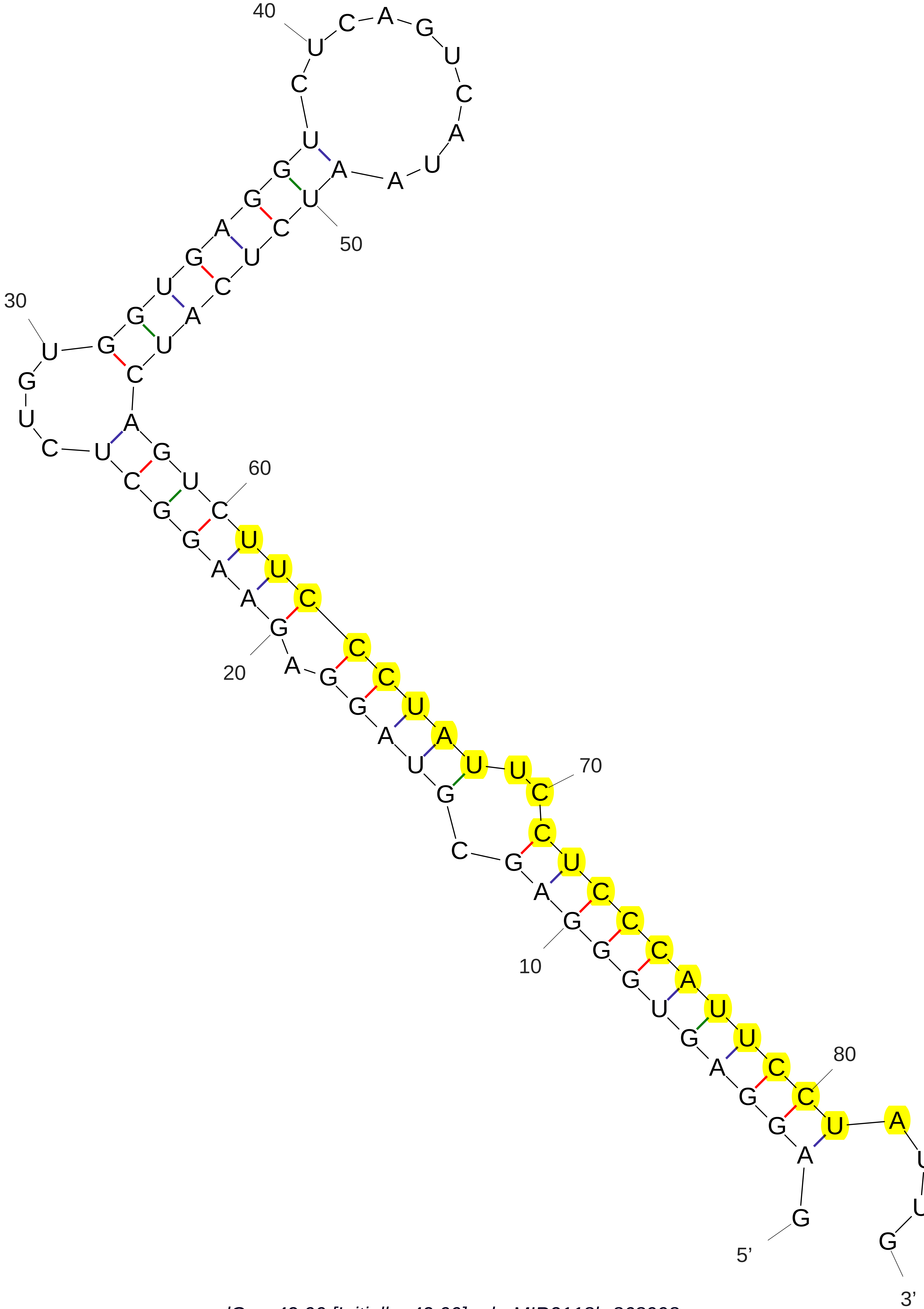

dG = -42.00 [Initially -42.00] pde-MIR2118b-868998

Output of sir_graph (©)
mfold_util 4.6

Created Tue Aug 16 22:27:48 2011

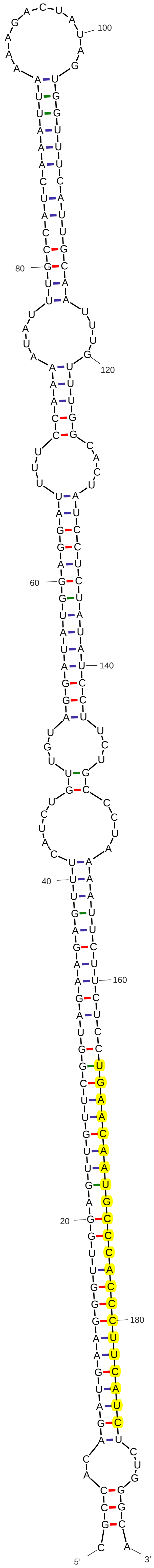

dG = -84.10 [Initially -84.10] pde-MIR3701

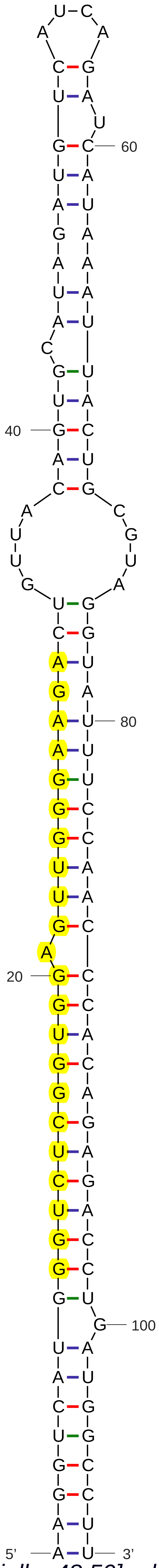

dG = -49.00 [Initially -48.50] pde-MIR3704

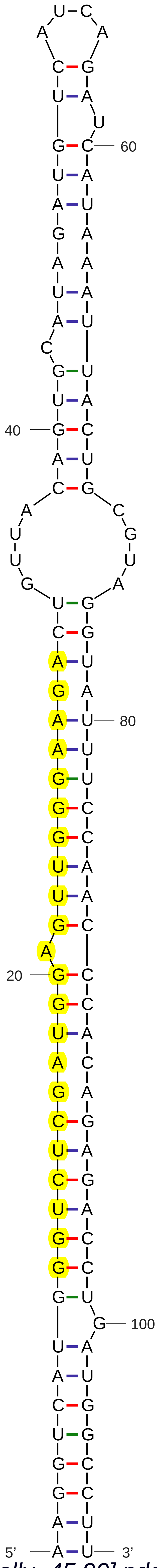

dG = -46.40 [Initially -45.90] pde-miR3704b

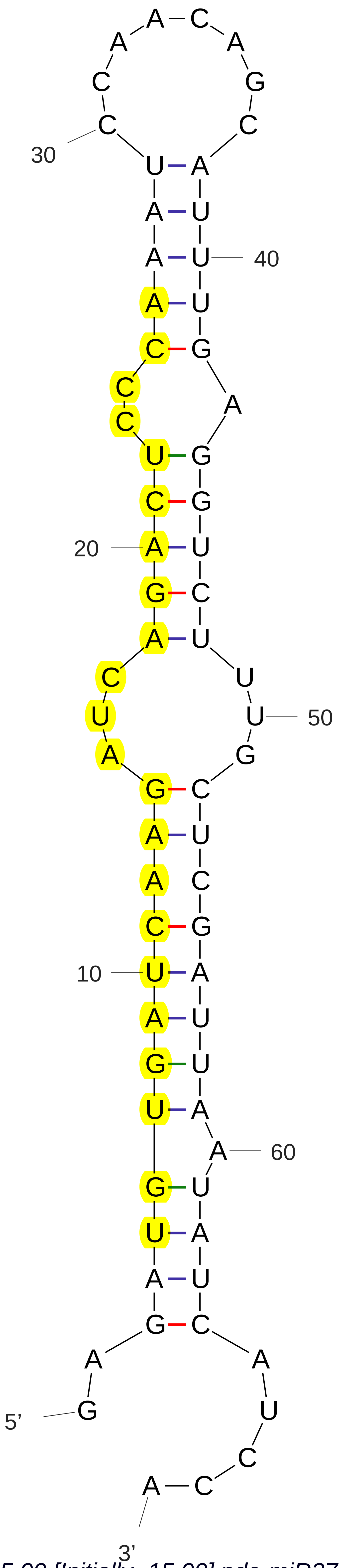

dG = -15.00 [Initially -15.00] pde-miR3712
