# Supplementary material for: Transcriptome-wide identification and characterization of miRNAs from Pinus densata
Source: BMC Genomics. 2012 Apr 6;13:132. doi: 10.1186/1471-2164-13-132 (PMC3347991; doi:10.1186/1471-2164-13-132)
Supplement: Additional file 4 — Predicted stem-loop structures of precursors containing P. densata microRNA sequences (red and blue). [file 1471-2164-13-132-S4.DOC]

**Additional file 4 Predicted stem-loop structures of precursors containing *P. densata* microRNA sequences (red and blue).**

**pde-miR159a**

(dG = -94.74)

UAAG UCA AUC - G- UUGUU

5’- CGGUAGAGCUCCUUUUGUACCAA AGGACUGUGCAAAAAUGAUCCGACUGCCGAUUUAUGC CUUCUGC CCUGCGA UGUUCG U

3’- GUCAUCUCGAGGGAAGUUUGGUU UCCUGUUGUGUUUUAGUUAUGUUGAGGGCGGAAUACG GAAGACG GGACGUU AUAAGC C

-CGG UGC --- - AG CUGAA

Loop 27 nt Loop 10 nt

**pde-miR162a**

(dG = -49.10)

UUU AA AUGU A G

5’- GCGA CAGACGCUUGGCAGCCUGGAUGCA GGGUUUACCGACC CC CGG C

3’- CGUU GUCUAAGGUUUGUUAGACCUACGU UCCAAAUAGCUGG GG GUC C

--- C- CUCU - G

**pde-miR166a**

(dG = -41.90)

GG - C UAGUCC U

5’- GGAAUGUUGUCUGGCUCGA GGC AUCCUGAAAA ACCCCCGG U

CCUUACUUCGGACCAGGCU CCG UAGGUUUUUU UGGGGUUU C

-- G - ------ U

**pde-miR166b**

(dG = -43.40)

GG A CGA

5’- GGAAUGUUGUCUGGCUCGAGGUCACUCAG UUUGAUGA \

3’- CCUUACUUCGGACCAGGCUGCAGUGAGUC GAGUUUCU U

-- - UAG

**pde-miR169a**

(dG = -48.80)

ACUAUUUAUUC AUC UUACCA UAG

5’- AGCCAAGGAUGACUUGCCUAG UUG GUAUCGGC \

3’- UCGGUUCCUGCUGAGUGGAUC GAC UAUAGUCG U

--------AAA ACU UUUAGC UAU

**pde-miR169b**

(dG = -79.50)

U UGAG C ACUAUU AUC UUACCA UAG

5’- UCAUCUG CUUGCAUG AGAGGCAGA UAUUCAGCCAAGGAUGACUUGCCUAG UUG GUAUCGGC \

3’- AGUAGAC GAUCGUAU UCUGCGUCU AUAAAUCGGUUCCUGCUGAGUGGAUC GAC UAUAGUCG U

- UCCA - AUUUUU ACU UUUAGC UAU

**pde-miR171a**

(dG = -55.30)

GAA CACG

5’- AGAAUGUGAUGUUGGCUAGGCUCAAUCGGAUUGUAACGCC G

3’- UCUUACACUAUAACCGUGCCGAGUUAGUCUAGUGUUCUGG A

CAA UUUA

**pde-miR390a**

(dG = -55.30)

UAAUGGUAUAAAGA CUUG

5’- AAUUAUGAAGCCCAGGAUGGAUAGCGCCAGCCCCA \

3’- UUAAUGUUUCGAGUCCUCCCUAUCGCGGGUGACGU A

------------CC UUAA

**pde-miR396a**

(dG = -43.28)

----- UCAU --- U

5’- UUUUCCCACGGCUUUCUUGAACUUC UCGGA ACUG U

3’- AAAAAGGUGCCGAAAGAACUUGAAG GGCCU UGAC A

AAUAU ---- GCG C

Loop 23 nt

**pde-miR482a**

(dG = -60.90)

UGAGAAGU UUU U CUUG AC- U CUUCA

5’- GAAGGGAUGUGU GUGGA GGGAGU AGGAGUGGGAG UAGGA AAGGCUG \

3’- CUUCCCUACACA CACUU CCGUUA UCCUUACCCUC AUCCU UUCUGAC U

----AACC --C - ---- CUC - CACUA

**pde-miR482b**

(dG = -42.90)

------A CUUG G- A CUGU UUCA

5’- UGUGGAUGGAAGU AGGAGUGGGAG GUAGG GAAGGCU GGUGAGGU G

3’- ACACUUCCCUCCG UCCUUACCCUC UAUCC CUUCUGA CUACUCUA U

CAAUGUA UUA- CU - ---- AUAC

**pde-miR482c**

(dG = -45.20)

AA - - U--- - UAU

5’- GGCCAAUGGCUUGCGAGG GUAGGAAAAGCU CAG GUGA UGA A

3’- CCGGUACCCGAACCCUCC CACCCUUUUUGA GUC CACU GCU U

-- U C UAGU C CUU

**pde-miR482d**

(dG = -46.50)

----------------- - - UUU -- UC-------- UU

5’- GU C AUGGGGUCUUUAGGC GGAGGAUUUGGAAAGG CUUAG AUUCUU U

3’- CA G UACUCCUGAUAUCCG CCUCCGCAACCUUUCC GAAUC UAGGAG A

GACGCUAGCCCACUGUA U U UAC AA UUUUAGCUUA CC

**pde-miR783**

(dG = -26.80)

- UGCAUU UUCA UAUUC

5’- UUCUUUUGAGGGAAGG GAGCUGGCG UCUU GA A

3’- AAGUAGAGUCCCUUUU CUUGGUCGU AGAA CU U

A UUCUU CCG UGCGU

**pde-miR946a**

(dG = -71.50)

- -C ------- - GAAA UU-- A- CU AUUA

5’- CAGAGU GUAUAGUUGUGGAUA AGAAGGGU UAGUAAAC GGUA ACCCAUUUA GUAU UCAUUGG GUUCGAG \

3’- GUUUGA CAUGUUAACACCUAU CUCUUCCCG CUCGUUUG CCGU UGGGUAAAU CAUA GGUAGCU UAAUGUC A

U C ACCAUGU U ---- UACU CC -- CACG

**pde-miR947**

(dG = -70.70)

- AAAGCA- CA GAA UU

5’- ACGCCUAAGGCGCAGCAGCAGAUUCUGAUAGAAGAC UCAGGC UUUGUUGUUGG GGUU UGC \

3’- UGCGGAUCCUUUGUCAUUGUCUAAGGCUACGUUCUG GGUCCG GAACAAUAACC CCAA ACG U

U CGGGGGG A- G-- GC

**pde-miR949a**

(dG = -41.80)

-C -A ---CU CG

5’- AGAGCUUCUCU GGAAUCAAAUGUGUCUUCCU UGAA \

3’- UCUCGAAGAGG CCUUAGGUUACCCGGAAGGA ACUU C

CC GC CACGC UC

**pde-miR949b**

(dG = -66.30)

C C- A AG

5’- AGAGCCUCUCCGGGAAUC AAUGCGCCUUCCUCUUGAACGC UUUCA CGCGC \

3’- UCCCGAAGGGGCCUUUAG UUAUGUGGAAGGAGGAUUUGCG AAAGU GCGUG A

- CA - UC

**pde-miR950a**

(dG = -40.90)

GUC U-- UC

5’- GAAGGUGAUCUUUACAUCUG CACGGUGGUUUAUUGU CA \

3’- CUUCCACGAGAAUAGUAGAC GUGCUACCAAAUAGUA GU G

GCC UUU UU

**pde-miR951**

(dG = -43.00)

CGU ACGUGGGUUUGCUUUACGUU GC- AUA

5’- GAAGCGAUGGUGUUCUUGA CUGGACC GG AUGA A

3’- UUUCGUUUCCACAAGAACU GACUUGG CC UACU A

ACU CG------------------ AUC AAC

**pde-miR952a**

(dG = -232.79)

GCGAGCTATCGAAGGAGA 213 nt

5’- GAACCAGUGGCGUAUUGAACAGAGCAUGCCAUUGGUGGAGU-------------

3’- CUUGGUAGUCGUUUAACUUGACUCGCACAGUAACCGCCUCC-------------

------------------ 213 nt

**pde-miR952b**

(dG = -214.40)

GCGAGCTATCGAAGGAGA 186 nt

5’- GAACCAGUGGCGUAUUGAACAGAGCAUGCCAUUGGUGGAGU-------------

3’- CUUGGUAGUCGUUUAACUUGACUCGCACAGUAACCGCCUCC-------------

------------------ 186 nt

**pde-miR952c**

(dG = -192.12)

GCGAGCTATCGAAGGAGA 182 nt

5’- GAACCAGUGGCGUAUUGAACAGAACAUGCCAUUGGUGGAGU-------------

3’- CUUGGUAGUCGUUUAACUUGACUCGCACAGUAACCGCCUCC-------------

------------------ 183 nt

**pde-miR1310**

(dG = -35.00)

AUUAGA A -- - G- CUCAA

5’- GGC UCG GGGGC GUAACGCCCUC ACCUAUU \

3’- CCG AGU CCUCG CGGCGUGGGAG UGGAUAA A

-----G - UA U AA AUUUC

**pde-miR1311**

(dG = -43.40)

-- - CGC A AC

5’- GUAGGAACAGGCGG ACUGGCAUAACUC CCAU UUUU U

3’- CAUCCUUACCCGCC UGACCGUUUUGAG GGUA GAGA G

GU U ACU - CU

**pde-miR1312a**

(dG = -41.50)

Loop 28 nt

- A- - A

5’- UCCCCCAAAACAUCCAUAUCGCUAU UCUCUCCUAAUUA GUUGAUUCAAAU U

3’- AGCGGCUUUUGUAGAUACAGCGGUA AGAGAGGUUUAAU CGUCUUUGUUUG U

U AA A A

**pde-miR1313**

(dG = -66.72)

Loop 13 nt

------------UCG UAU CG-- A C-- ------- ---- CAGCGUUUCGCCA A

5’- UGAUGG UCUACCACUGAAAUUAUUGUU AAAU ACAC UGCAAU CCACCGUGGUAUG GCAUCCUU AACUA \

3’- GCUACC AGAAGGUGACUUUAAUAACAA UUUA UGUG ACGUUG GGUGGUACGGUAC CGUUGGGA UUGAU C

UACUCAGAAGGUCUA UUC CCUA - AGU ------- ACAA CC----------- C

Loop 23 nt

**pde-miR1314a**

(dG = -42.30)

GUCA -- U - UU

5’- UGUU GGU AGAUGAAGGAAUUCUUC AACAUUAGAGGCCGAUGUGGAG \

3’- ACGA CCA UCUCCUUUUGUAAGAGG UUGUAAGCUCCGGCCAGUUCUC A

---- AU - A GU

**pde-miR1448**

(dG = -46.50)

----------------- - - UUU -- UC-------- UU

5’- GU C AUGGGGUCUUUAGGC GGAGGAUUUGGAAAGG CUUAG AUUCUU U

3’- CA G UACUCCUGAUAUCCG CCUCCGCAACCUUUCC GAAUC UAGGAG A

GACGCUAGCCCACUGUA U U UAC AA UUUUAGCUUA CC

**pde-miR2118a**

(dG = -46.50)

----------------- - - UUU -- UC-------- UU

5’- GU C AUGGGGUCUUUAGGC GGAGGAUUUGGAAAGG CUUAG AUUCUU U

3’- CA G UACUCCUGAUAUCCG CCUCCGCAACCUUUCC GAAUC UAGGAG A

GACGCUAGCCCACUGUA U U UAC AA UUUUAGCUUA CC

**pde-miR2118b**

(dG = -42.00)

G--- C- A CUGU CUCA

5’- AGGAGUGGGAG GUAGG GAAGGCU GGUGAGGU G

3’- UCCUUACCCUC UAUCC CUUCUGA CUACUCUA U

GUUA CU - ---- AUAC

**pde-miR3701**

(dG = -84.10)

C AC- CAUCU UGU UUU- AUAU AAAGA

5’- GCC AGAUGAAGGGUUGGAGUUGUUCGGUAGAAGAGUUU GU AGGAUAUGGAGGAU CCAAA UUGCCAUCAAAUUA \

3’- CGG UCUACUUCCCACCCGUAACAAGUCCUCUUCUUAAA CG UCCUAUAUCUCCUA GGUUU AACGUUACUUUGGU C

A GUC AUCC- UCU UCAC GUUU GAUAU

**pde-miR3704a**

(dG = -49.00)

- A GUUA C - A

5’- AAGGUCAU GGGUCUCGGUGG GUUGGGAAGACU CAGUG AUAGAUG UC U

3’- UUCCGGUA UCCAGAGACACC CAACCUUUAUGG GUCAU UAAAUAC AG C

G - AUGC - U A

**pde-miR3704b**

(dG = -46.40)

- A GUUA C - A

5’- AAGGUCAU GGGUCUCGAUGG GUUGGGAAGACU CAGUG AUAGAUG UC U

3’- UUCCGGUA UCCAGAGACACC CAACCUUUAUGG GUCAU UAAAUAC AG C

G - AUGC - U A

**pde-miR3712**

(dG = -15.00)

---GA - AUC CC CCA

5’- GAUG UGAUCAAG AGACU CAAAU A

3’- CUAU AUUAGCUC UCUGG GUUUA C

ACCUA A GUU A- CGA
