# Supplementary material for: Transcriptome-wide identification and characterization of miRNAs from Pinus densata
Source: BMC Genomics. 2012 Apr 6;13:132. doi: 10.1186/1471-2164-13-132 (PMC3347991; doi:10.1186/1471-2164-13-132)
Supplement: Additional file 5 — Conserved mature miRNAs without hairpin structure in P. densata. [file 1471-2164-13-132-S5.DOC]

**Additional file 5 Conserved mature miRNAs without hairpin structure in *P. densata*.**

| **miRNA gene** | **miRNA sequence (5'-3')** | **Arm** | **Length**  **(nt)** | **Homologous** |
| --- | --- | --- | --- | --- |
| pde-miR156 | UGACAGAAGAGAGAGAGCAC | 5' | 20 | osa-miR156k |
| pde-miR399 | UGCCAAAGGAGAAUUGCCC | 3' | 19 | osa-miR399a |
| pde-miR414 | UCGUCCUCAUCAUCAUCGUCC | 3' | 21 | osa-miR414 |
| pde-miR948 | ACAGGCUUUGUGGGAUCCGG | 5' | 20 | pta-miR948 |
| pde-miR1171 | UGGAAUGGAGUGGAGUGGAGUGG | 5' | 23 | cre-miR1171 |
| pde-miR1309 | UGAUGGCCUUUUUGAAGGACA | 5' | 21 | pta-miR1309 |
| pde-miR1316 | UCCAUACACAAACCAUUGGAA | 3' | 21 | pta-miR1316 |
