# Supplementary material for: Transcriptome-wide identification and characterization of miRNAs from Pinus densata
Source: BMC Genomics. 2012 Apr 6;13:132. doi: 10.1186/1471-2164-13-132 (PMC3347991; doi:10.1186/1471-2164-13-132)
Supplement: Additional file 6 — Predicted targets of P. densata miRNAs and their putative functions. [file 1471-2164-13-132-S6.DOC]

**Additional file 6 Predicted targets of *P. densata* miRNAs and their putative functions.**

| **miRNA family** | **Target function** | **Target^a^** |
| --- | --- | --- |
| pde-MIR156 | SBP-domain protein | Singletons9853 (0)  Singletons54865 (0.5)  Singletons74798 (0.5) |
|  | Unknown | Singletons59747 (3) |
| pde-MIR159 | ATP binding protein/protein kinase | Singletons16733 (3) |
|  | Unknown | Singletons11093 (3)  Singletons18913 (3) |
| pde-MIR169 | Unknown | Singletons34481 (1.5)  Singletons83401 (3) |
| pde-MIR396 | Unknown | Singletons74936 (3) |
| Pde-MIR399 | Unknown | Singletons18651 (2.5) |
| pde-MIR414 | Nuclear assembly factor 1 | Singletons14673 (1) |
|  | 8-oxoguanine DNA glycosylase | Singletons84399 (1.5) |
|  | ELMO domain-containing protein | Singletons17918 (2) |
|  | Ribosomal protein S6 family protein | Singletons10112 (2.5) |
|  | Chromodomain helicase DNA binding protein | Singletons71684 (2.5) |
|  | Histone methyltransferase (SUVH2) | Singletons899 (3) |
|  | Unknown | Singletons14434 (0)  Singletons9902 (1.5)  Singletons73033 (1.5)  Singletons68655 (2)  Singletons14835 (2.5)  Singletons14998 (2.5)  Singletons63778 (2.5)  Singletons72110 (3)  Singletons82759 (3)  Singletons83063 (3) |
| pde-MIR482 | RNA binding / calmodulin binding / endonuclease/ nucleic acid binding / protein binding (CPSF30) protein | Singletons19513 (3) |
|  | Histone deacetylase | Singletons7264 (3) |
|  | Peptidyl-prolyl cis-trans isomerase | Singletons84503 (3) |
| pde-MIR946 | Unknown | Singletons13919 (2.5) |
| pde-MIR947 | Unknown | Singletons58451 (2.5) |
| pde-MIR948 | Unknown | Singletons15872 (1)  Singletons48550 (2)  Singletons4502 (2.5)  Singletons69101 (2.5) |
| pde-MIR949 | Unknown | Singletons16231 (3) |
| pde-MIR950 | Unknown | Singletons10016 (3) |
| pde-MIR951 | Cytoplasmic ribosomal protein S13-like | Singletons70237 (3) |
|  | Unknown | Singletons52549 (3)  Singletons8494 (3) |
| pde-MIR952 | Unknown | Singletons9891 (3) |
| pde-MIR1171 | Unknown | Singletons973 (3)  Singletons83957 (3) |
| pde-MIR1309 | Unknown | Singletons18506 (2.5)  Singletons18578 (2.5)  Singletons6246 (3)  Singletons17805 (3) |
| pde-MIR1312 | Argonaute/Zwille-like protein | Singletons18790 (3) |
| pde-MIR1313 | Flagellin-sensing 2-like protein | Singletons46417 (3) |
|  | Unknown | Singletons5839 (2.5)  Singletons76069 (2.5)  Singletons36440 (3)  Singletons81039 (3)  Singletons83703 (3) |
| pde-MIR1314 | P-glycoprotein | Singletons66322 (2.5) |
| pde-MIR1316 | Brassinosteroid insensitive 1-associated receptor kinase | Singletons12055 (3) |
|  | Unknown | Singletons2669 (1.5)  Singletons5667 (1.5)  Singletons226 (2.5) |
| pde-MIR1448 | Unknown | Singletons12934 (3)  Singletons65538 (3)  Singletons72472 (3) |
| pde-MIR2118 | Disease resistance protein | Singletons12934 (2) |
|  | Anion exchanger family protein | Singletons8179 (3) |
| ^a^ All predicted miRNA targets with penalty scores (shown in parentheses) of three or less are listed. | | |
