# Supplementary material for: Transcriptome-wide identification and characterization of miRNAs from Pinus densata
Source: BMC Genomics. 2012 Apr 6;13:132. doi: 10.1186/1471-2164-13-132 (PMC3347991; doi:10.1186/1471-2164-13-132)
Supplement: Additional file 7 — Primers used for amplifying P. densata miRNAs and their targets. [file 1471-2164-13-132-S7.DOC]

**Additional file 7 Primers used for amplifying *P. densata* miRNAs and their targets.**

| **Primer name** | **Sequence (5'-3')** |
| --- | --- |
|  | **For qPCR** |
| pde-miR159a | CCTTTGGTTTGAAGGGAGCTCTA |
| pde-miR166a | GTCGGACCAGGCTTCATTCC |
| pde-miR171a | TGATTGAGCCGTGCCAATATC |
| pde-miR390a | AAGCCCAGGATGGATAGCGCCAA |
| pde-miR396a | TCCCACGGCTTTCTTGAACTT |
| pde-miR946a | CAGCCCTTCTCCTATCCACAA |
| pde-miR950a | CCTCTGGTCCACGGTGGTTTAT |
| pde-miR1311 | TCAGAGTTTTGCCAGTTCCGC |
| pde-miR1313 | GCTACCACTGAAATTGTTGTTCG |
| pde-miR1314 | CCGGCCTCGAATGTTAGGAGA |
| pde-5s rRNA | GGA AGTCCTAGTGTTGCACCCTC |
|  | **For subcloning** |
| pde-miR159aF | TTTTTTTAGGTATGTTCATATGGT |
| pde-miR159aR | CCAGTAGAGCTCCCTTCAAACCAA |
| pde-miR166aF | GAGGACGAGGAGGGCAAGAAGTAG |
| pde-miR166aR | GGAATGAAGCCTGGTCCGACGTCA |
| pde-miR166bF | GACATTGAAGGTGTCCTCGTTGTG |
| pde-miR166bR | GGAATGAAGCCTGGTCCGACGGCA |
| pde-miR169aF | AAAAAAAATTCATTTGAAATGGCC |
| pde-miR169aR | GTGGAAGCCCAGAGAATTTGCATT |
| pde-miR171aF | GCAGATGGTACAGTGTTGTAAACG |
| pde-miR171aR | AATGGAATGGAATGGAGTGGATGA |
| pde-miR482aF | GGCGAGAAGGCCTTATTGTTTTGT |
| pde-miR482aR | TAGGGATGTGTAGTGAAGGCCAAT |
| pde-miR482bF | TTTGTTGATTTTGAGAAGGGATCT |
| pde-miR482bR | CGGAGACATTTTTATTTGCCTGAA |
| pde-miR482dF | GGGTCTTTAGGCTTTGGAGGATTT |
| pde-miR482dR | CGATCGGGTGACATGTACAATGAG |
| pde-miR783F | CCACTTTCAAAAGTACCTCGGCCT |
| pde-miR783R | AGGGACCAACAGGATTGATGCGCT |
| pde-miR946aF | TTTCTACGCAGGATCCTAGGGTTT |
| pde-miR946aR | CAAACTAGTACAATTGTGGATAGGA |
| pde-miR947F | GGAAGCAGTTAAGCGCAGGCTGCT |
| pde-miR947R | GCCTTATCGGATCAAATTCACCAG |
| pde-miR949aF | CAGAGCTTCTCTAGGAATCAAATG |
| pde-miR949aR | GGAGAGCTTCTCCCGGGAATCCAA |
| pde-miR949bF | GCGCGTGAAGGAGAGCCTCTCCG |
| pde-miR949bR | GAATCTGGAACAAAACGGTCAGTT |
| pde-miR950aF | GCCGATTCAAAATGATGGGTAGAT |
| pde-miR950aR | ATTAAGATAAAATCAACTTAACTTTAACC |
| pde-miR951F | GAAGCGATGGTGTTCTTGACGTCT |
| pde-miR951R | CATGTAAATGAAAGCAAAGGTGTT |
| pde-miR952aF | GCGAGCTATCGAAGGAGAGAACCA |
| pde-miR952aR | GAACCATCAGCAAATTGAACTGAG |
| pde-miR1310F | ATTAGAGGCATCGGGGGCGTAAC |
| pde-miR1310R | CGGCTCAATGGAGCAGCCGCACC |
| pde-miR1311F | TGGCCGCCTCTGCTTATGGGCTT |
| pde-miR1311R | TAAAGGCACCGAGCATATCGAATT |
| pde-miR1312aF | GCGGTAGGGGGATTCGGTAACGCC |
| pde-miR1312aR | CCTGCAGCGTATTGGAGTACAGCAT |
| pde-miR1313aF | TCGTGATGGTATTCTACCACTGAA |
| pde-miR1313aR | TCTTCCAGATCGATGGAAGTCTTC |
| pde-miR3701F | GTGAAAAGTTCTCGTGGAAAGCTC |
| pde-miR3701R | AAAAACAAAAAACTAGCACAAAAG |
| pde-miR3704F | CGGAGACATTTTTATTTGCCTGAA |
| pde-miR3704R | TTTGTTGATTTTGAGAAGGGATCTG |
| pde-miR3712F | AGAATAAATCCTTATCACGAGATG |
| pde-miR3712R | TGGATGATATTAATCGAGCAAAGA |
| pde-actinF | AGAAATCCAGCCCCTTGTA |
| pde-actinR | CCCCATACCAACCATCACA |
|  | **For RLM-5' RACE** |
| P11093P1 | TAGTGAACCCAGTGAAGCGAAAAT |
| P11093P2 | ACTGTCCTTCACCAAACCATCAAC |
| P7264P1 | TTGAATCTTTGAGCAACACGCACA |
| P7264P2 | CTTTGAGCAACACGCACAATGACT |
| P83401P1 | ATTTCACGCAGTTCTTCTTTTTCA |
| P83401P2 | CAGTTCTTCTTTTTCACCTGGCTT |
| P10015P1 | TCCAGCGGTGAAGAGATATTTGAA |
| P10015P2 | GCGGTGAAGAGATATTTGAAGTTG |
